# Responses of Murine and Human Macrophages to Leptospiral Infection: A Study Using Comparative Array Analysis

**DOI:** 10.1371/journal.pntd.0002477

**Published:** 2013-10-10

**Authors:** Feng Xue, Xinghui Zhao, Yingchao Yang, Jinping Zhao, Yutao Yang, Yongguo Cao, Cailing Hong, Yuan Liu, Lan Sun, Minjun Huang, Junchao Gu

**Affiliations:** 1 Beijing Friendship Hospital, Capital Medical University, Beijing, China; 2 Beijing Tropical Medicine Research Institute, Beijing, China; 3 Beijing Key Laboratory for Research on Prevention and Treatment of Tropical Diseases, Beijing, China; 4 Beijing Institute of Biotechnology, Beijing, China; 5 Division of Parasitic Vaccines, Institute for Biological Product Control, National Institutes for Food and Drug Control, Beijing, China; 6 School of Life Sciences, Tsinghua University, Beijing, China; 7 Department of Neurobiology, Capital Medical University, Beijing, China; 8 Beijing Key Laboratory of Major Brain Disorders, Beijing Institute of Brain Disorders, Beijing, China; 9 College of Veterinary Medicine, Jilin University, Changchun, China; Institut Pasteur, France

## Abstract

Leptospirosis is a re-emerging tropical infectious disease caused by pathogenic *Leptospira* spp. The different host innate immune responses are partially related to the different severities of leptospirosis. In this study, we employed transcriptomics and cytokine arrays to comparatively calculate the responses of murine peritoneal macrophages (MPMs) and human peripheral blood monocytes (HBMs) to leptospiral infection. We uncovered a series of different expression profiles of these two immune cells. The percentages of regulated genes in several biological processes of MPMs, such as antigen processing and presentation, membrane potential regulation, and the innate immune response, etc., were much greater than those of HBMs (>2-fold). In MPMs and HBMs, the caspase-8 and Fas-associated protein with death domain (FADD)-like apoptosis regulator genes were significantly up-regulated, which supported previous results that the caspase-8 and caspase-3 pathways play an important role in macrophage apoptosis during leptospiral infection. In addition, the key component of the complement pathway, C3, was only up-regulated in MPMs. Furthermore, several cytokines, e.g. interleukin 10 (IL-10) and tumor necrosis factor alpha (TNF-alpha), were differentially expressed at both mRNA and protein levels in MPMs and HBMs. Some of the differential expressions were proved to be pathogenic *Leptospira*-specific regulations at mRNA level or protein level. Though it is still unclear why some animals are resistant and others are susceptible to leptospiral infection, this comparative study based on transcriptomics and cytokine arrays partially uncovered the differences of murine resistance and human susceptibility to leptospirosis. Taken together, these findings will facilitate further molecular studies on the innate immune response to leptospiral infection.

## Author Summary

Although pathogenic *Leptospira* is not an obligate intracellular pathogen, recent studies have shown that phagocytosis and innate immunity play important roles in leptospirosis. The *Leptospira*-macrophage interaction is a common model used to elucidate the initial response in leptospiral infection. Our previous research has shown that there is little difference in the transcriptomics of pathogenic *Leptospira* infecting murine or human macrophage cell lines. Contrarily, in this study, we observed significant differences of murine and human primary macrophages infected by *L. interrogans* as shown in several processes, such as antigen processing and presentation, Toll-like receptor signaling pathway and innate immune response, complement and coagulation cascades, expression of major cytokines and chemokines, etc. These results suggested that different immune responses explain the major disparities in the murine and human *Leptospira*-macrophage infection models. This study added to the former leptospiral transcriptomics research on the *Leptospira*-macrophage interaction model and laid a foundation for further investigation in the pathogenesis of leptospirosis.

## Introduction

Leptospirosis is an important tropical infectious disease around the world, particularly in humid tropical and subtropical countries [1], [2]. The causal agents include several pathogenic *Leptospira* spp., of which the highly virulent strains (e.g. *Leptospira interrogans*) chronically infect reservoir hosts (e.g. wild rodents) without causing severe symptoms; however, *L. interrogans* acutely infects humans and causes severe organ failure and mortality in some individuals. The urine released from a chronically infected reservoir host contains a high concentration of leptospiral cells, which can survive and replicate in moist soil and water for a long time before infecting the next subject. The pathogen can infect humans through mucous membranes or abrasions in the skin, penetrate into the blood stream, and rapidly diffuse into the liver, lung, kidney, and other organs [1]. The clinical symptoms are complex, including hemorrhage, diarrhea, jaundice, severe renal impairment, aseptic meningitis, etc. [2]. Multiple components of the pathogen, such as lipopolysaccharide (LPS) [3], peptidoglycans [4], glycolipoproteins [5], lipoproteins [6], and transmembrane or outer membrane proteins (OMPs) [6], are involved in induction of the host immune response and cytokine secretion.

Although previous research has shown that humoral immunity is important in leptospirosis [7], [8], the role of innate immunity in controlling leptospiral infection has recently been uncovered in cell infection models and animal infection models. Phagocytosis is key to the early defenses of hosts to bacterial infection, while pathogenic *Leptospira* can escape complement attack and phagocytosis upon infection [9], [10]. In *in vitro* cell infection models, unlike nonpathogenic *L. biflexa*, pathogenic *L. interrogans* can rapidly attach and invade macrophages [11], [12] and induce apoptosis [13]. Pathogenic *Leptospira* have also been found to survive and replicate in human macrophages but are killed in murine macrophages [14]. The LPSs of pathogenic *Leptospira* activate human macrophages only through Toll-like receptor 2 (TLR2) [3], while they activate murine macrophages through both TLR2 and TLR4 [15]. In addition, the cytokine expression differs between mouse and human macrophages as revealed by *in vitro* cell infection models [16]. These previous studies suggest that the different innate immune responses of murine and human macrophages correlate with the differences of murine resistance and human susceptibility to leptospirosis.

The expression patterns of cytokines and chemokines in different animal infection models have also been comparatively analyzed to reveal the mechanisms of anti-*Leptospira* immunity and identify predictors of leptospirosis [17], [18], [19]. Though hamsters [19], [20] and the TLR4-deficient murine models [10], [21] that mimic human acute leptospirosis, were appropriately used, the immune responses of acute infections in animal models may not fully resemble those in humans. The immune responses demonstrated in human primary cells infected by pathogenic *Leptospira* may improve our understanding of human leptospirosis. In addition, the approaches used to study immune responses have been limited to specific genes and pathways, and the kinetic signaling transduction and molecular activation process of host immunity remain largely unknown [17]. In this study, we applied gene expression microarrays and cytokine arrays to comparatively analyze responses of murine and human macrophages to leptospiral infection and identify more activated inflammatory genes and signaling pathways in this *in vitro* cell infection model.

## Materials and Methods

### Ethics statement

Dunkin-Hartley ICO:DH (Poc) guinea pigs and BALB/c mice were bred and maintained under specific pathogen-free conditions in the animal facilities of Capital Medical University. All animal experiments complied with the Regulations for the Administration of Affairs Concerning Experimental Animals in China, the Chinese Standards on Experimental Animals, and the Manual for Bacterial Inspection (GB/T 14926.42-2001). Animal protocols were approved by the Animal Ethics Review Committee of Capital Medical University (Approval number: CCMU-AE20110129). Written informed consents were signed by the participants, and the protocols were approved by the Ethics Review Committee of Capital Medical University (Approval number: CCMU-PE20110212).

### Bacterial strains

The standard cultures of pathogenic *L. interrogans* serovar Lai strain Lai (56601) and saprophytic *Leptospira biflexa* serovar Patoc strain Patoc I (Paris, 651505) were obtained from the Division of Parasitic Vaccines, Institute for Biological Product Control, National Institute for Food and Drug Control (NIFDC), which is also the National Center for Medical Culture Collections of China. The strains were cultivated at 28°C in Ellinghausen-McCullough-Johnson-Harris (EMJH) liquid medium for passage. The virulence of *L. interrogans* was preserved by iteratively infecting specific pathogen-free Dunkin-Hartley ICO: DH (Poc) guinea pigs (10 to 12 days old; each weighing less than 150 g). The *L. interrogans* was recovered from the kidneys of the infected guinea pigs, washed three times using autoclaved phosphate-buffered saline (PBS), and cultured for three generations in EMJH medium to exclude possible sources of animal components. *L. interrogans* and *L. biflexa* were respectively proliferated in 50 ml of EMJH mediums for use in the experimental infections. The close relative of *L. interrogans* [22], nonpathogenic *L. biflexa*, was used as a control to verify the pathogenic *Leptospira*-specific gene regulations and cytokine expressions in this study.

### Host cells

Murine peritoneal macrophages (MPMs) were isolated from male BALB/c mice (6 to 8 weeks old) by washing the peritoneal cavities with cold RPMI 1640 medium. Macrophages were seeded in 75-cm^2^ tissue culture flasks (Corning, NY, USA), and the cell numbers were counted in a hemocytometer. The cells were cultured in RPMI 1640 medium (supplemented with 10% heat-inactivated fetal bovine serum, 100 µg/ml penicillin, and 100 µg/ml streptomycin) for 2 h at 37°C under a humidified atmosphere containing 5% CO_2_. After preincubation, non-adherent cells were removed gently by washing with 0.01 M PBS (pH 7.4), and the purity of the remaining macrophages was tested by Wright's staining. Human peripheral blood monocytes (HBMs) were isolated from twelve healthy donors using standard Ficoll-Hypaque gradient centrifugation as previously described [23]. The monocytes were seeded and counted according to the above-mentioned protocol for MPMs and then preincubated overnight at 37°C under a humidified atmosphere containing 5% CO_2_. After preincubation, the monocytes were thoroughly washed with autoclaved PBS to remove non-adherent cells and then continuously incubated for 5 days in medium containing 500 U/ml granulocyte-macrophage colony stimulating factor (GM-CSF; eBioscience, CA, USA) to differentiate the cells into macrophages.

### Cell infection models

The MPM and HBM cultures were washed three times with autoclaved PBS, renewed with new medium without antibiotics, and further cultured for 12 h before infection. Leptospiral cells (*L. interrogans* or *L. biflexa*) were harvested by centrifugation at 8,000 *g* for 10 min at 20°C, and then washed twice with PBS. The bacterial pellets were resuspended in RPMI 1640 medium (supplemented with 10% heat-inactivated FBS) at 37°C to a final concentration of 10^8^ bacteria/ml. Ten ml of bacterial suspension (10^9^ bacteria) was added to 10^7^ cells (bacteria∶cell = 100∶1, the total culture medium volume was 20 ml), and the tissue culture flasks were centrifuged at 2500 *g* for 15 min to synchronize the infection. Then, the infection models were incubated at 37°C under a humidified atmosphere containing 5% CO_2_. Host cell samples were collected at three intervals (1, 2, and 4 h), respectively, in the cell infection models; and three biological replicates were performed for each sample. The high efficiency of leptospiral infection were revealed by indirect immunofluorescence and examined by confocal microscopy. Briefly, the lysosomes of the infected MPMs and HBMs (for 1-h) were labeled with 1∶1000 diluted lysosome marker Lamp1 Abs (Invitrogen, CA, USA), then labeled with Alexa Fluor 488-conjugated F(ab′)_2_ anti-rabbit Ab (Invitrogen, CA, USA). While, the leptospiral cells were labeled with 1∶200 diluted rat antiserum against *L. interrogans* strain Lai or *L. biflexa* strain Patoc I (NIFDC, Beijing, China), and then labeled with Texas Red-conjugated F(ab′)_2_ anti-rat IgG Ab (Invitrogen, CA, USA). The fluorescence signals were captured by an Olympus FV1000 laser scanning confocal fluorescence microscope. Previous cytokine mRNA kinetic expression studies have shown that most cytokine transcripts can be detected as early as 1 h after infection in an animal infection model [17], indicating that rapid gene regulation of innate immune response can be detected within a 4-h period in this study. The MPM and HBM cultures not infected by *L. interrogans* or *L. biflexa* were used as negative controls.

### Total RNA isolation and cDNA synthesis

At sample harvesting, MPM and HBM cultures were washed three times using autoclaved PBS to remove leptospiral cells. The total RNA was extracted using TRIzol reagent (Invitrogen, CA, USA), then purified using the RNeasy Mini Kit (QIAGEN, Hilden, Germany) with on-column DNase digestion (QIAGEN, Hilden, Germany) according to the RNeasy Mini handbook. RNA quantity and integrity were determined using an RNA 6000 Nano Laboratory-on-a-Chip kit and a Bioanalyzer 2100 (Agilent Technologies, CA, USA). For each sample, approximately 10 µg of total RNA was mixed with 600 ng of oligo d(T)primers (TaKaRa, Otsu, Japan) and denatured at 65°C for 5 min. Then, the first strand cDNA was synthesized using 2 µl (400 U) of SuperScript III reverse transcriptase (Invitrogen, CA, USA), according to the protocol recommended by the manufacturer. The double-stranded cDNA (ds cDNA) sample was then synthesized using the second Strand Synthesis section of the M-MLV RTasecDNA Synthesis Kit (TaKaRa, Otsu, Japan), according to the manufacturer's instructions. Following RNase H (Invitrogen, CA, USA) and RNase A (Ambion, TX, USA) digestion for 1 h, the ds cDNA sample was purified using a QIAquick PCR Purification Kit (QIAGEN, Hilden, Germany), according to theQIAquick Spin handbook.

### Microarray hybridization and data analysis

The Phalanx OneArray mouse whole genome microarray (Phalanx, Hsinchu, Taiwan) containing 31,802 highly sensitive 70-mer sense-strand polynucleotide probes, including 29,922 mouse gene probes and 1880 experimental control probes, was used. In addition, the Phalanx OneArray human whole genome microarray containing 32,050 highly sensitive 60-mer sense-strand polynucleotide probes, including 30,968 human gene probes and 1082 experimental control probes, was used. The microarray experiments were performed by SinoGene Scientific Co., Ltd., Beijing, China, according to the microarray manufacturer's instructions. Briefly, the ds cDNA templates were labeled using Amersham Mono-functional Cy5 CyDye and hybridized to the OneArray whole genome microarray with Phalanx hybridization buffer using cover slides. After overnight hybridization at 50°C, nonspecific binding targets were washed away using three different washing steps. The slides were dried using centrifugation and scanned using a GenePix 4000B microarray scanner (Molecular Devices, CA, USA). The Cy5 fluorescence intensities of each spot were analyzed using GenePix Pro 6.0 software (Molecular Devices, CA, USA). The signal intensity of each spot was corrected by subtracting background signals in the immediate surroundings. Spots with flag <0 and a signal-to-noise ratio <3, and the control probes were filtered out. Spots that passed the above-mentioned criteria were normalized using quantile normalization, according to the manufacturer's recommendation, and tested for differential expression. The differentially expressed genes were further analyzed using Agilent GeneSpring GX software (version 10.0) and CapitalBio MAS (Molecule Annotation System, version 3.0, CapitalBio, Beijing, China). Considering that primary cells are a mixture of several related cell types with limited purity [24], regulated genes with more than 3-fold (P<0.05) up-regulation or down-regulation were defined as significantly regulated genes in this report. The regulations induced during the 4-h leptospiral infection (same regulation trends at 1 h, 2 h, and 4 h) were counted in the statistical analysis. Meanwhile, some instantaneous regulations at the 2-h time points were also included if needed.

### Quantitative real-time reverse transcription polymerase chain reaction (qRT-PCR)

To validate the microarray data, the highly regulated genes, differentially expressed genes between MPMs and HBMs, six randomly selected genes, and sixteen highly regulated and differentially expressed genes from a new batch of RNA samples were quantitatively analyzed using qRT-PCR. The cell infection model, RNA extraction, and cDNA synthesis were performed according to the microarray cDNA synthesis protocol. In addition, MPMs and HBMs infected by *L. biflexa* (bacteria∶cell = 100∶1) were designed as controls to verify that the differentially expressed genes in leptospiral infection were pathogenic *Leptospira*-specific regulations. The primers were designed using Premier software version 5 (Premier Biosoft International, CA, USA) (**Tables S1**, **S2**, and **S3**). RT reaction mixtures contained 0.5 µg of total RNA, 100 ng of random hexamer primers (TaKaRa, Otsu, Japan), 0.5 µl (100 U) of Superscript III reverse transcriptase (Invitrogen, CA, USA), and 500 µM concentrations each of dATP, dCTP, dGTP, and dTTP. After denaturation at 65°C for 5 min, the samples were incubated at 50°C for 1 h, followed by 10 min at 70°C to synthesize the first strand cDNA. Samples of 30 ng of cDNA were mixed with 25 µl of 2×SYBR Green PCR Master Mix (TaKaRa, Otsu, Japan). Assays were performed in triplicate with the ABI PRISM model 7500 sequence detection instrument. A relative quantification method was used to calculate the regulation folds in different infection samples. For each target gene, an amplicon was obtained using the qRT-PCR primers and standard RT-PCR, then the concentration of the purified amplicon was determined using Qubit 2.0 fluorometer (Invitrogen, CA, USA). The gradient dilutions of the amplicon were used as control templets in qRT-PCR to construct a gene-specific standard curve. The templet quantifications in different infection samples were finally obtained by comparison with the standard curve. The melting curves were used to evaluate whether the accumulation of SYBR Green-bound DNA was gene specific.

### Cytokine array immunoblot assay

Semi-quantitative protein membrane arrays (RayBiotech, GA, USA, Cat. No. AAM-CYT-3-8 for MPMs, and AAH-CYT-6-8 for HBMs) containing 62 mouse or 60 human cytokine antibodies were used to verify cytokine and chemokine expression of macrophages at the protein level in this study. The membrane arrays were spotted with capture antibodies to various cytokines and chemokines including tumor necrosis factor alpha (TNF-alpha), TNF-beta, interleukin (IL)-1beta, IL-10, IFN-gamma, MIP-1alpha, MCP-1, MIP-1beta, IP-10, et al. Briefly, 5×10^5^ MPMs and HBMs were infected with 5×10^7^ *L. interrogans* or 5×10^7^ *L. biflexa* cells (bacteria∶cell = 100∶1) for 4 h, respectively. The final culture medium volume of each sample was 1 ml in a block of a 24-well plate (Corning, NY, USA), which corresponded to the culture medium volume used in the previous infection model for the microarray assay. MPMs and HBMs that were not infected were used as negative controls. The host cells were washed three times using autoclaved PBS, and the proteins were extracted using cell lysis buffer (RayBiotech, GA, USA, with protease inhibitor). The membrane arrays were incubated with 2 ml of cell lysates for the immunoblot assay. Captured cytokines and chemokines were visualized using immunohistochemistry following the manufacturer's instructions. Three biological replicates were performed.

## Results

### Validation of infection efficiency and RNA integrity of macrophages

Unlike the heat-killed *Leptospira* used in prior infection models [16], a live and strong virulent *L. interrogans* strain was used to infect primary macrophages to establish the cell infection model in this study. The infection times were strictly limited to a short period (4-h), and only a few live leptospiral cells (less than 1%) survived in macrophages. Most of the extracellular bacteria, along with apoptotic and dead host cells, were removed by repeated PBS washes. The infection efficiencies were checked by indirect immunofluorescence of the *Leptospira*-containing phagosomes. The high infection ratio (*Leptospira*∶cell = 100∶1) guaranteed that almost all the macrophages were infected by *Leptospira* (**Figure S1**). To verify the integrity and purity of total RNA, RNA samples were determined using a Bioanalyzer 2100 high performance capillary electrophoresis (HPCE) instrument. Bacterial 23S and 16S RNA bands were not detected by HPCE (**Figure S2**).

### Verification of microarray data, highly regulated genes, and differentially expressed genes

The microarray data were validated using qRT-PCR. The transcriptional levels of the six randomly selected genes were determined using qRT-PCR performed on a new batch of RNA samples. No PCR amplification was detected in the negative controls. The qRT-PCR values of the infected MPMs and HBMs at 1-, 2-, and 4-h time points were plotted against the corresponding microarray data values, respectively. The high correlation coefficient values (R^2^≥0.85) indicated that the microarray signal represented by multiple oligonucleotide probes was valid for transcriptomics research (**Figures S3** and **S4**).

The gene regulation folds of sixteen highly regulated genes and differentially expressed genes between MPMs and HBMs, such as TLR1, LPS-binding protein, NF-kappa B inhibitor alpha, IL-10, TNF-alpha, IL-6, CCL5, CXCL9, MIP-1 alpha, MIP-1 beta, MIP-2, C3, CASP8 and FADD-like apoptosis regulator, IL-1-alpha, IL-1-beta, and BCL-2 genes, were further verified by qRT-PCR. As a control, the regulation folds of these genes in MPMs and HBMs infected by *L. biflexa* were also calculated to confirm that the different regulations of eight genes (i.e. TLR1, LPS-binding protein, IL-10, MIP-1 alpha, MIP-1 beta, MIP-2, C3, and BCL-2) between MPMs and HBMs were pathogenic *Leptospira*-specific regulations (**Figure S5**).

### Verification of cytokine and chemokine protein expression

**Figure 1:**
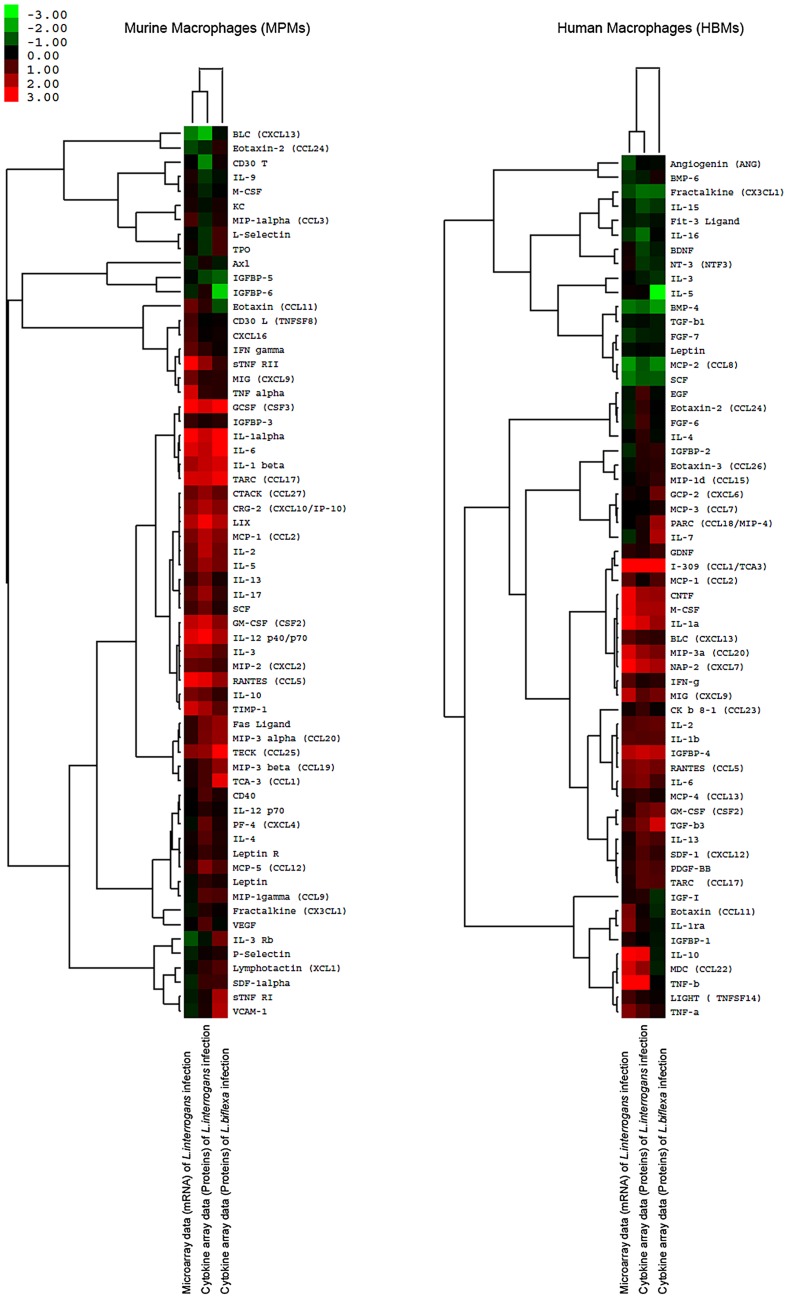
Comparison of cytokine regulation folds of murine peritoneal macrophages (MPMs) and human peripheral blood monocytes (HBMs) at mRNA and protein levels. Hierarchical cluster analyses of the average microarray data and the average cytokine array data of 62 cytokines of murine peritoneal macrophages (MPMs) and 60 cytokines of human peripheral blood monocytes (HBMs) were performed using Cluster3.0 software and visualized by using TreeView software. MPMs and HBMs were infected by *L. interrogans* for 4-h or infected by *L. biflexa* for 4-h, respectively.

The protein expression levels of the 62 murine and 60 human cytokines as well as the chemokines of the macrophages infected by *L. interrogans* were verified by using immunoblot assays (i.e. RayBiotech semi-quantitative protein membrane arrays). The quantitative data of the microarrays and the cytokine arrays were comparatively analyzed using hierarchical cluster analysis of Cluster 3.0 software and visualized by TreeView software (**Figure 1**). At 4-h intervals, most of the cytokine and chemokine protein expression levels corresponded to their mRNA expression levels. However, the protein expression fold changes of several of the most highly regulated cytokines, such as GCSF (CSF3) (19.31-fold at mRNA level, and 5.78-fold at protein level) and TNF alpha (5.62-fold at mRNA level, and 1.46-fold at protein level) in MPMs, and IL-10 (26.14-fold at mRNA level, and 7.21-fold at protein level), IL-1alpha (11.03-fold at mRNA level, and 5.74-fold at protein level), and TNF-beta (15.89-fold at mRNA level, and 9.06-fold at protein level) in HBMs, were less than the mRNA fold changes. These results may be due to the feedback or post-transcriptional regulations of these cytokines. As a control, the cytokine expression of MPMs and HBMs infected by *L. biflexa* were also calculated to verify that the different regulations between MPMs and HBMs were pathogenic *Leptospira*-specific regulations (**Figure 1**). Nine cytokines and chemokines (i.e. CXCL13, CCL11, IL-13, IL-17, IL-3Rb, CCL19, CCL1, CCL25, and VCAM-1) were differentially regulated in MPMs infected by *L. interrogans* and *L. biflexa*. And, ten cytokines and chemokines (i.e. CXCL6, CCL1, IL-10, IL-16, IL-5, IL-7, CCL22, CCl18, TGF-b3, and TNF-b) were differentially regulated in HBMs infected by *L. interrogans* and *L. biflexa*.

### Transcriptomics and pathway analysis

**Figure 2:**
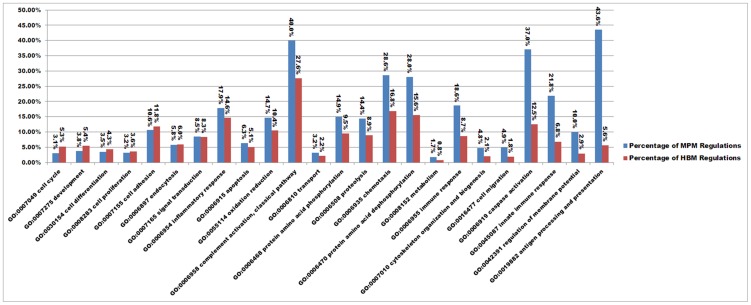
The differences of regulated biological processes of murine peritoneal macrophages (MPMs) and human peripheral blood monocytes (HBMs) infected by *L. interrogans*. The persistent regulations during the 4-h leptospiral infection, not the instantaneous regulations at the 1-h and the 2-h time points, were included in the statistical analysis using CapitalBio MAS (Molecule Annotation System version 3.0) software. The values above the bars show the percentages of total regulations, including up-regulations and down-regulations, of each GO biological process.

The microarray project was deposited in the DDBJ as BioProject ID PRJDB733, and the microarray data was deposited in the NCBI-GEO as ID GSE45170. In total, 1891 up-regulated genes and 431 down-regulated genes in MPMs, and 1932 up-regulated genes and 629 down-regulated genes in HBMs were induced during the 4-h leptospiral infection (same regulation trends at 1 h, 2 h, and 4 h), which did not include the instantaneous regulations at the 1-h or the 2-h time points. Based on the Gene Ontology (GO) biological process, the percentage of regulated genes in each GO biological process (i.e. the number of regulated genes divided by the number of total genes in the pathway) was calculated to study the differences of gene regulation in MPMs and HBMs infected by *L. interrogans*. The percentages of regulated genes in eight biological processes of MPMs were significantly greater than those of HBMs (>2-fold), such as GO:0019882, antigen processing and presentation (7.87-fold); GO:0042391, regulation of membrane potential (3.47-fold); GO:0045087, innate immune response (3.11-fold); GO:0006919, caspase activation (2.98-fold); GO:0016477, cell migration (2.62-fold); GO:0007010, cytoskeleton organization and biogenesis (2.29-fold); GO:0006955, immune response (2.18-fold); and GO:0008152, metabolism (2.12-fold) (**Figure 2**). These differences suggested that HBMs were less activated, which could eventually contribute to *L. interrogans* evading the host immunity and favor establishment of the infection.

**Figure 3:**
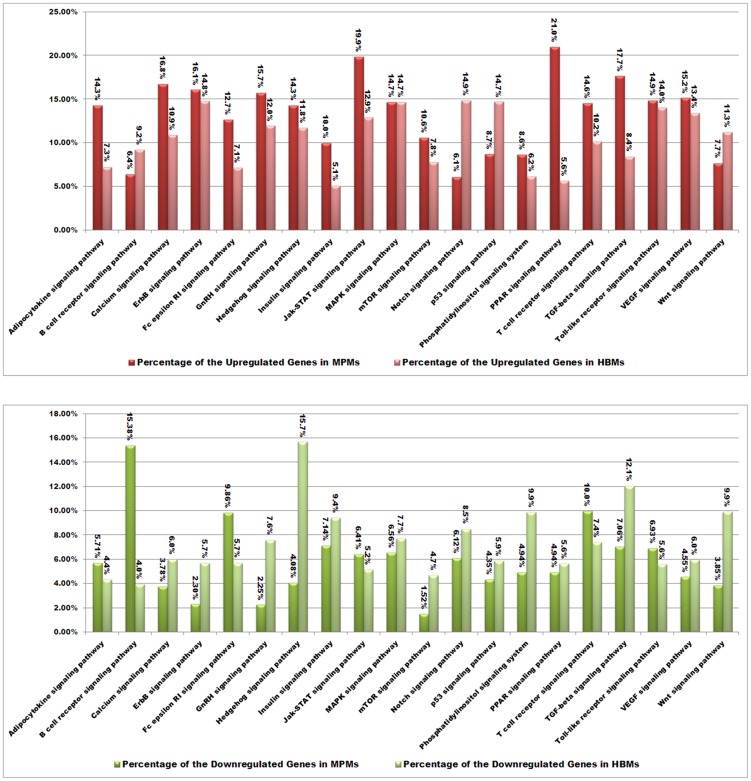
The differences of up-regulated and down-regulated signaling pathways of murine peritoneal macrophages (MPMs) and human peripheral blood monocytes (HBMs) infected by *L. interrogans*. The persistent regulations during the 4-h leptospiral infection, not the instantaneous regulations at the 1-h and the 2-h time points, were included in the statistical analysis using CapitalBio MAS (Molecule Annotation System version 3.0) software. The values above the bars show the percentages of total regulations, including up-regulations and down-regulations, of each KEGG signaling pathway.

Although signal transduction often occurs at the level of phosphorylation, not at the level of transcription, the time series gene regulation analysis revealed a series of signaling factor changes in this study. The percentage of up-regulated and down-regulated genes in each Kyoto Encyclopedia of Genes and Genomes (KEGG) signaling pathway (i.e. the number of up- or down-regulated genes divided by the total genes in the pathway) was used to evaluate the significance of gene regulation of the signaling pathway. For the up-regulations, the percentages of up-regulated genes in the PPAR signaling pathway and the TGF-beta signaling pathway of MPMs were much greater than those of HBMs (>2-fold). The percentage of up-regulated genes in the Notch signaling pathway of HBMs was much greater than that of MPMs (>2-fold). For the down-regulations, the percentage of down-regulated genes in the B cell receptor signaling pathway of MPMs was much greater than that of HBMs (>2-fold). The percentages of down-regulated genes in the ErbB, GnRH, Hedgehog, mTOR, phosphatidylinositol, and Wnt signaling pathways of HBMs were much greater than those of MPMs (>2-fold) (**Figure 3**), which suggested that the cell differentiation and development signals that were closely related to MAPK and PI3K pathways may be less activated in HBMs. In summary, these KEGG pathway statistics primarily revealed that the signaling pathways of MPMs showed more up-regulation and less down-regulation than those of HBMs, and the signal transductions of MPMs may be more active than those of HBMs during *L. interrogans* infection. The main regulated infection-related biological processes and pathways are discussed in detail below.

### TLR signaling pathway and innate immunity

The host TLR signaling pathway plays an important role in identifying pathogens through their pathogen-associated molecular patterns (PAMPs). At least 11 TLRs have been identified so far in immune cells. Most TLRs can be constitutively expressed in macrophages or macrophage cell lines [25], [26], and some of their expressions can be induced during infection [26]. Several leptospiral components, such as LPS, lipoproteins, and OMPs, have been verified to activate host cells by TLRs [3], [6], [15]. Leptospiral LPS only induces immune response through TLR2 in human but through both TLR2 and TLR4 in mouse [15]. Although the stimulating activity of leptospiral LPS in peripheral monocytes is at least 1000-fold less than that of the LPS of *E. coli* [10], the TLR2 signal induced by leptospiral LPS may be very important in human leptospirosis, as shown by the extraordinarily high expression level of TLR2 in human monocytes [26]. In addition, leptospiral OMPs and lipoproteins also induce immune response through the TLR2 signaling pathway [6]. Previous research has shown that the leptospiral outer membrane has a relatively complex antigen profile compared to other pathogenic spirochetes [27]. Only the OMPs of pathogenic *Leptospira*, not those of saprophytic *Leptospira*, can mediate early inflammation in proximal tubule cells [6].

**Figure 4:**
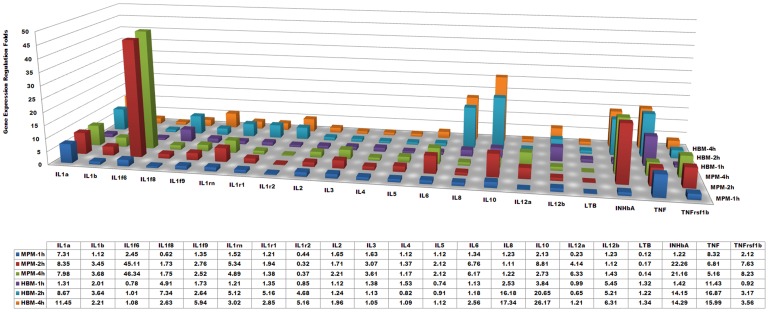
Comparisons of major regulated inflammatory cytokines and receptors in murine peritoneal macrophages (MPMs) and human peripheral blood monocytes (HBMs) infected by *L. interrogans*. MPM-1 h, -2 h, and -4 h show the gene expression fold changes of the leptospiral-infected MPMs at 1-, 2-, and 4-h, respectively; HBM-1 h, -2 h, and -4 h show the gene expression fold changes (Ln(microarray folds)) of the leptospiral-infected HBMs at 1-, 2-, and 4-h, respectively. Average fold changes are listed in the affiliated table below.

The genes involved in the TLR signaling pathway showed significant regulations during *L. interrogans* infection. The major pro-inflammatory effective cytokine genes, such as the genes of TNF-alpha, IL-1-beta, etc., were up-regulated in both MPMs and HBMs (**Figure 4**). Though no significant regulation of TLR2/4 was observed during the 4-h leptospiral infection, the different expressions of the cytokines may partially be due to the different regulation of the other adaptors and receptors. For example, the TLR1 gene (*tlr1*) was up-regulated in MPMs, but it was down-regulated in HBMs. Considering that TLR1 heterodimerizes with TLR2 to recognize triacetylated lipoproteins, and also participates in the recognition of leptospiral LPS in human cells [15], the different regulation may be related with the differential activation by leptospiral lipoproteins and LPS. The LPS-binding protein gene (*lbp*) was only persistently down-regulated in MPMs infected by *L. interrogans*. These two different regulations between MPMs and HBMs were pathogenic *Leptospira*-specific regulations which was not happened in MPMs or HBMs infected by *L. biflexa* (**Figure S5**). In addition, myeloid differentiation primary response factor (MyD88), the key adaptor downstream of the TLR pathways, was down-regulated in HBMs; Two key factors of the MyD88-independent pathway, TRAM (TIRP/TICAM-2) and TRIF (TICAM-1), were only down-regulated in HBMs. while the Toll-IL-1 receptor domain-containing adapter protein (TIRAP) controlling the activation of MyD88-dependent pathways downstream of TLR-4 [28], was down-regulated in MPMs. Previous research has shown that the OMPs from pathogenic *Leptospira* and the purified LipL32 lipoprotein all up-regulated TLR2 expression [6]. However, the TLR2 gene expression in this study was not regulated in MPMs nor in HBMs; hence, this result may be due to down-regulation of the OMPs previously revealed in renal tubule and urine as well as our cell infection models [29], [30].

In addition, the genes involved in the nucleotide-binding domain and leucine-rich repeat (NLR) containing signaling pathway showed few regulations during leptospiral infection. The NLRP10 (NACHT, leucine rich repeat and PYD containing 10) gene was moderately up-regulated in MPMs. The NLRP14 (NACHT, leucine rich repeat and PYD containing 14) gene was persistently up-regulated, while the NLRP13 (NACHT, leucine rich repeat and PYD containing 13) gene was persistently down-regulated in HBMs. Though pathogenic *Leptospira* spp. is an extracellular pathogen, the intracellular signaling pathway should not be ignored due to the intracellular life cycle [14] and the NLRP3-dependent cytokine secretion [31].

### NF-kappa B signaling pathway

NF-kappa B regulates the expression of many genes involved in immune and inflammatory response [32]. NF-kappa B heterodimers can migrate from cytoplasm to nucleus, and regulate different sets of target genes [33]. NF-kappa B is an anti-apoptosis factor because it can up-regulate the expression of cell death inhibitors; thus, inhibition of NF-kappa B promotes cell death [34]. It has been reported that NF-kappa B activation and p38 phosphorylation can be induced by *Leptospira* infection or exposure to partially purified leptospiral lipoproteins in microglial cells [35], and induced by leptospiral LPS and lipoproteins in human and murine macrophage cell lines [3], [15]. In this study, the up-regulation folds of the LPS-inducible NF-kappa B inhibitor alpha gene (*nfkbia*) and zeta gene (*nfkbiz*) in HBMs were significantly greater than those in MPMs, and the NF-kappa B 1 gene (*nfkb1*) was only slightly up-regulated in HBMs. This gene regulation difference suggested that induction of the NF-kappa B signaling cascade in HBMs may be less than that in MPMs. The other signaling pathways, such as the p53 and MAPK signaling pathways, etc., showed a few regulation changes in our cell infection models. However, the phosphorylation signaling cascades in these pathways deserve more attention in future leptospiral infection studies.

### Inflammatory cytokines and receptors

Inflammatory cytokines are immunomodulatory proteins that help the host mount an immune response to diverse inflammatory processes. It has been reported that hamsters that died from leptospirosis had significantly greater expression levels of both pro- and anti-inflammatory mediators in comparison to the survivors [19]. The inflammation induced by pathogenic *Leptospira* is is always less than that induced by other Gram-negative pathogens [36]. Previous research has shown that *L. interrogans* can induce pro-inflammatory cytokines, such as TNF-alpha, IL-6, etc., in mouse peritoneal macrophages [10], [16]. Though high expression levels of pro-inflammatory cytokines from macrophages were verified to correlate with the clearance of pathogenic *Leptospira* [10], recent research has also shown that susceptible TLR-deficient mice infected with *Leptospira* died from both elevated levels of pro-inflammatory cytokines and high bacterial loads [21]. Furthermore, pathogenic *Leptospira* can induce production of type 1 cytokines involved in cellular immunity in a hamster infection model [17]. As expected, the genes of the major cytokines, including TNF-alpha, IL-1 alpha/beta, IL-10, and inhibin beta-A, etc., were significantly up-regulated both in MPMs and HBMs during leptospiral infection; while the expression levels of IL-2 and IFN-gamma did not change significantly in MPMs and HBMs (**Figure 4**).

TNF-alpha induces monocytes to secrete other cytokines, such as IL-1, IL-6, and IL-8, which are essential for controlling infection and cleaning LPS from blood, and is also involved in sepsis and tissue lesions [37], [38]. Previous research has shown that *L. interrogans* induces greater TNF-alpha levels than those stimulated by *Borrelia garinii* and *Treponema pallidum*, but less than that activated by *E. coli* LPS in isolated rat liver macrophages (Kupffer cells). The greater expression of TNF-alpha was mainly induced by leptospiral LPS, which indicated that TNF-alpha up-regulation may be related to severe hepatitis during leptospirosis [39]. In addition, other clinical research has shown that TNF-alpha is closely related to severe nephritis during human leptospirosis [40]. In this study, the expression levels and the fold change of TNF-alpha expression in HBMs were all greater than those in MPMs (>2-fold, P<0.05), both at mRNA and protein levels (**Figures 1** and **S5**). However, in the *L. biflexa* infection control, the up-regulation folds of TNF-alpha protein expression in HBMs were also significantly higher than those in MPMs (>2-fold, P<0.05) (**Figure 1**), which indicated that the different regulation of TNF-alpha during *L. interrogans* infection was not pathogenic *Leptospira*-specific. The high expression level of TNF-alpha in HBMs may be related to the severe pathological symptoms of human leptospirosis. Interestingly, possibly due to the divergent expression of TNF-alpha, IL-6 was only up-regulated in MPMs and IL-8 was up-regulated in HBMs (**Figure 4**). These results were primarily consistent with the previous comparative cytokine analysis in different macrophages that the IL-6 level in mouse cells rose more rapidly than it did in human cells [16].

A recent study showed that leptospiral LPS synergizes with glycolipoprotein to produce IL-1-beta [31]. In this study, IL-1 alpha/beta and its receptor (IL-1R, ICE) were up-regulated in both MPMs and HBMs infected by *L. interrogans* and *L. biflexa*, both at mRNA and protein levels (**Figures 1** and **S5**). The upper activation pathways of IL-1 alpha/beta include the activation of pro-caspase-1, a cytosolic protease, to become caspase-1 after inflammasome activation, and activated caspase-1 causes the proteolytic processing of pro-IL-1-beta, resulting in maturation and secretion of IL-1-beta [41].

IL-10 is a multifunctional anti-inflammatory cytokine that plays an important role in limiting inflammatory response and preventing tissue damage [42]. It can be secreted by macrophages, and it suppresses the release and function of other factors, such as IL-1beta, IL-6, TNF-alpha, IL-12, etc. [34]. TNF-alpha can induce expression of IL-10, while IFN-gamma and IL-10 itself can inhibit the production of IL-10. Our microarray data showed that the expression of TNF-alpha during *L. interrogans* infection was greater than that of IL-10 after 1-h, which was consistent with the inductive effect of TNF-alpha on IL-10. However, the expression levels and regulation patterns of IL-10 were dramatically different in MPMs and HBMs infected by *L. interrogans* (**Figures 1**, **4** and **S5**). The IL-10 expression in MPMs was detectable at a low level and not further up-regulated after 4-h, while IL-10 was persistently up-regulated (more than 20-fold) in HBMs, both at mRNA and protein levels. However, in the *L. biflexa* infection control, the IL-10 protein expressions in MPMs and HBMs were not induced at the 4-h time point (**Figures 1** and **S5**). These results were different from the previous reports that leptospiral glycolipoprotein induces IL-10 production of human peripheral blood mononuclear cells [5]. In addition, the IL-10 receptor (IL-10R) gene was down-regulated in MPMs, which may further reduce the functional role of IL-10 in MPMs. A previous study on borrelial infection has shown that IL-10 is differentially expressed in macrophages from different murine models, suggesting that it may contribute to the control of inflammation in Lyme disease [43]. Therefore, it was reasonable to presume that the low expression level of IL-10 in MPMs may contribute to a high inflammatory response, which may help mice to reduce the leptospiral burden during infection. Furthermore, the IL-10/TNF-alpha ratio has been proposed as a prognosis indicator in sepsis during leptospirosis [44]. This ratio reflects a persistent secretion of IL-10 at a later stage in septic patients. In this study, the IL-10/TNF-alpha ratio in macrophages within this short time period may not be relevant to the outcome in mouse and human infection [19].

The interferon-gamma (IFN-γ) expression level has been verified to be very low in patients who have leptospirosis [16], [45]. This cytokine, which is not produced by macrophages, is mainly produced by activated T helper 1 cells, T cells, and natural killer (NK) cells [46]. Therefore, it was not unexpected to find that IFN-gamma in MPMs and HBMs infected by *L. interrogans* or *L. biflexa* was not up-regulated (**Figure 1**). A previous *in vitro* study using human whole blood stimulated by heat-killed *L. interrogans* showed that the production of IFN-gamma is partially controlled by IL-12 secreted by macrophages [47]. In our live *Leptospira* cell infection model, the IL-12a gene of MPMs and the IL-12b gene of HBMs were persistently up-regulated during *L. interrogans* infection. However, up-regulation of the IL-12b gene of HBMs may not further induce IFN-γ expression in CD4+ T cells during human leptospirosis, since IL-10 in HBMs was also up-regulated and the high-level of IL-10 could suppress the immune response.

IL-6 is a pleiotropic cytokine that influences antigen-specific immune responses and inflammatory reactions, and it is always treated as the best marker for severity of infectious stress [48]. Previous research has shown that leptospiral heat stable components other than LPS can stimulate mouse macrophage IL-6 expression [16], and leptospiral infection can induce IL-6 secretion in a mouse model [10]. In this study, IL-6 was up-regulated in MPMs (5-fold at mRNA level and 3-fold at protein level), but it remained unchanged in HBMs, during *L. interrogans* infection. Therefore, it is debatable whether IL-6 should be used as a predictor in the early diagnosis of human acute leptospirosis. In the *L. biflexa* infection control, IL-6 was also only up-regulated in MPMs, which indicated that the different regulation of IL-6 in MPMs and HBMs may not be a pathogenic *Leptospira*-specific pattern (**Figures 1** and **S5**).

### Chemokines and receptors

**Figure 5:**
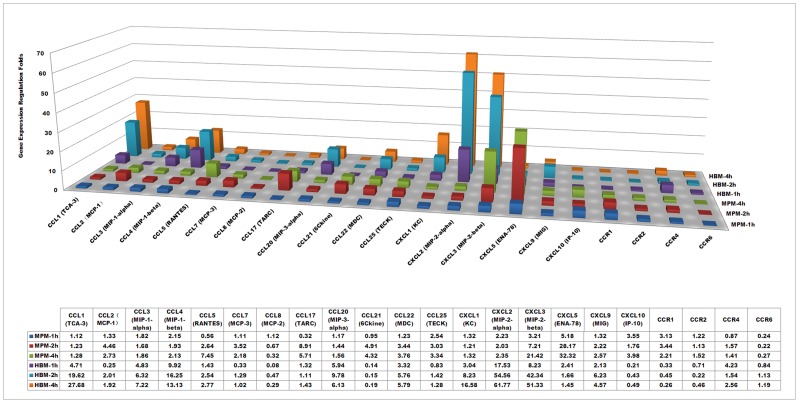
Comparisons of major regulated chemokines and receptors in murine peritoneal macrophages (MPMs) and human peripheral blood monocytes (HBMs) infected by *L. interrogans*. MPM-1 h, -2 h, and -4 h show the gene expression fold changes of the leptospiral-infected MPMs at 1-, 2-, and 4-h, respectively; HBM-1 h, -2 h, and -4 h show the gene expression fold changes (Ln(microarray folds)) of the leptospiral-infected HBMs at 1-, 2-, and 4-h, respectively. Average fold changes are listed in the affiliated table below.

Chemokines are a type of chemotactic protein that can attract leukocytes to the sites of infection. More than 40 chemokines have been identified in human, most of which are a group of structurally related cytokines involved in immune responses [49]. Previously, pathogenic *Leptospira* induced different chemokine profiles in resistant BALB/c and susceptible C3H/HeJ mice models during a two-week infection; hence, these results indicate that the distinct chemokine profiles may be related to the different outcomes in chronic and acute leptospiral infections [18]. In this study, significantly different chemokine regulations between MPMs and HBMs infected by *L. interrogans* were also revealed by microarray analyses (**Figure 5**) and partially by cytokine array analyses (**Figure 1**). The major difference in regulation between MPMs and HBMs was that six genes, including chemokine (C-C motif) ligand 5 (CCL5), chemokine (C-C motif) ligand 17 (CCL17, TARC), chemokine (C-C motif) ligand 25 (CCL25, TECK), chemokine (C-X-C motif) ligand 5 (CXCL5, ENA-78), chemokine (C-X-C motif) ligand 10 (CXCL10, IP-10), and chemokine (C-C motif) receptor 1 (CCR1), were only up-regulated in MPMs. However, compared with the protein expressions of the *L. biflexa* infection control (**Figures 1** and **S5**), these regulations were not pathogenic *Leptospira*-specific chemokine differences. While, eight genes, including chemokine (C-C motif) ligand 1 (CCL1), chemokine (C-C motif) ligand 3 (CCL3, MIP-1alpha), chemokine (C-C motif) ligand 4 (CCL4, MIP-1beta), chemokine (C-C motif) ligand 20 (CCL20), chemokine (C-X-C motif) ligand 1 (CXCL1), chemokine (C-X-C motif) ligand 2 (CXCL2, MIP-2), chemokine (C-X-C motif) ligand 9 (CXCL9, Mig), and chemokine (C-C motif) receptor 4 (CCR4), were only up-regulated in HBMs, in which the different mRNA or protein regulations of MIP-1 alpha, MIP-1 beta, MIP-2 should be pathogenic *Leptospira*-specific chemokine differences (**Figures 5** and **S5**). Though pronounced chemotactic gene regulations, including up-regulations and down-regulations, were observed in MPMs (**Figure 5**), the gene expressions of the major chemokines, such as MIP-1-alpha/beta, and MIP-2 were only significantly up-regulated in HBMs, which possibly reflects that HBMs would attract leukocytes to the sites of leptospiral infection more efficiently.

Unexpectedly, our result that MIP-1-alpha was not up-regulated in MPMs at both mRNA and protein levels seemed to be contradictory to previous animal infection models [18], [50], which showed that increases of MIP-1-alpha may contribute to host resistance to *Leptospira* infection in resistant BALB/c and Oncins France 1 mice. A reasonable explanation is that the expression levels of MIP-1-alpha can vary depending on the tissue and time after infection [18], so the expression of MIP-1-alpha in macrophages within 4-h may not resemble those in various animal tissues after infection for several days. Moreover, the significant up-regulation of MIP-2 in HBMs may correlate to the severity and progression of human leptospirosis.

### Complement and coagulation cascades

The complement system consists of a large population of plasma and membrane proteins that can be produced by macrophages, intestinal epithelial cells, liver and spleen cells, etc., and it plays important roles in both innate and adaptive immunity during infection [51]. Previous *in vitro* studies have shown that the complement system of healthy serum can kill saprophytic *Leptospira*, but not pathogenic *Leptospira* [52], and it is also indispensable for the phagocytosis of human polymorphonuclear leukocytes [53]. Recently, pathogenic *Leptospira* have been shown to be resistant to complement-mediated killing due to the fact that the pathogen can directly disrupt the complement system by capturing its components (e.g. factor H, C4BP, etc.) [54], [55], [56], [57]. In this study, the central component involved in both the classical and alternative pathways, the complement component 3 (C3) gene [58], was only up-regulated persistently in MPMs during *L. interrogans* infection (**Figure S5**). This different regulation was a pathogenic *Leptospira*-specific pattern. However, the negative complement regulator of the lectin and classical pathways, the C4BP gene, was also up-regulated by more than 10-fold in MPMs, while the C4BP-alpha gene was slightly down-regulated in HBMs. In addition, the C4B gene was only down-regulated in MPMs. This inconsistency made it difficult to understand the complement-mediated functions in MPMs. In summary, the complement components of host macrophages were easily regulated during the early stage of leptospiral infection, and they may be related with complement function and immune activation.

### Antigen processing and presentation

Macrophages are an important antigen-presenting cell (APC) of host immunity, although the antigen presentation of macrophages is less efficient than that of dendritic cells (DCs) [59]. Considering that murine macrophages kill and degrade leptospiral cells more efficiently than do human macrophages [14], the different regulations in the antigen processing and presentation pathways of MPMs and HBMs should be easily detected. As mentioned above, the percentage of regulated genes involved in antigen processing and presentation of MPMs was much greater than that of HBMs (**Figure 2**). In MPMs infected by *L. interrogans*, the cathepsin L gene and two histocompatibility-2 locus genes (T region locus 24 and Q region locus 10) were up-regulated; while another four histocompatibility-2 locus genes (class II antigen A alpha, class II antigen E alpha and beta, and O region beta locus) and an Ia-associated invariant chain gene were down-regulated. In HBMs infected by *L. interrogans*, a nuclear transcription factor Y alpha gene and an IFN-alpha-8 gene were up-regulated; while a regulatory factor X-associated protein gene, a proteasome (prosome, macropain) activator subunit 1 (PA28 alpha) gene, a major histocompatibility complex class I, E gene, a CD4 antigen (p55), an IFN-alpha-6 gene, and a regulatory factor X, 5 (influences HLA class II expression) gene were down-regulated. The genes involved in antigen processing and presentation pathways in MPMs and HBMs were mainly down-regulated, and the down-regulations in HBMs were more significant than those in MPMs. Taken together, these results suggested that antigen processing and presentation in HBMs was weaker than that in MPMs.

### Apoptosis

Pathogen invasion and internalization of bacterial components into a target cell by binding to a cell receptor can cause pathogen-induced apoptosis [60]. The apoptosis effect often occurs during the early stage of an infectious disease, and it can contribute to efficient colonization and diffusion of pathogens, initiation of inflammation in hosts, or defensive reactions in hosts [61]. It seems that macrophages are particularly susceptible to pathogen-induced apoptosis [62]. Previous research has revealed that pathogenic *Leptospira* can induce apoptosis in mouse macrophages by invasion or in hepatocytes by noninvasive mechanisms [11], [63]. In addition, macrophage apoptosis occurs through caspase-8 and caspase-3 pathways [13].

In general, the genes involved in this pathway showed robust up-regulations both in MPMs and HBMs. Especially, the caspase-8 and Fas-associated protein with death domain (FADD)-like apoptosis regulator genes were significantly up-regulated, which supported previous caspase-8 and -3 pathway results [13]. The upper signaling pathway of caspase-8 and the FADD-like pathway seem to be closely related to TNF-alpha and its receptor (TNF-R1), since the genes of TNF-alpha (Tnf), TNF (ligand) super family member 9 (Tnfsf9), TNF receptor super family member 1b (Tnfrsf1b), TNF receptor super family member 9 (Tnfrsf9), and TNF receptor-associated factor 1 (Traf1) in MPMs, and the genes of TNF super family member 2 (Tnfsf2), Tnfsf9, and Tnfrsf1b were all up-regulated in HBMs.

In addition to the highly consistent apoptosis of MPMs and HBMs, there were some differentially regulated genes. The caspase-3, caspase-7, and Bcl2-like 1 genes were only up-regulated in MPMs; while MYD88 and colony-stimulating factor 2 receptor beta 1 (low-affinity, granulocyte-macrophage) genes were only down-regulated in HBMs. Interestingly, the main anti-apoptotic gene, *bcl-2*, was only down-regulated in MPMs, which was also a pathogenic *Leptospira*-specific regulation (**Figure S5**). A previous report has shown that *bcl-2* is down-regulated and apoptosis is increased in macrophages after infection with *Mycobacteria bovis* BCG [64]. In addition, another BCL-2 family member, BCL2-related protein A1, was also significantly up-regulated in HBMs (>8-fold), which suggested that HBMs may antagonize cell apoptosis during the early stage of infection. In conclusion, these results were primarily consistent with previous results that MPMs and HBMs were all induced to become apoptotic by leptospiral infection, the apoptosis of MPMs occurred earlier than that of HBMs, and the proportion of early apoptotic cells in MPMs was significantly higher than that in HBMs during the 4-h leptospiral infection [14].

### Extracellular matrix (ECM)

Pathogenic *Leptospira* can bind to the host cells by protein interactions between ECM components and leptospiral surface proteins [65], [66], [67], [68], [69], [70], [71], [72], [73], [74], which should be closely related with the pathogenesis of leptospirosis. Recent studies also have shown that the OMPs of pathogenic *Leptospira* can induce ECM accumulation through a TGF-beta1/Smad-dependent pathway [75]. However, in our live *Leptospira* infection model, the up-regulations of ECM components, synthesis enzymes, and degrading enzymes made it difficult to confirm the ECM accumulation during infection (**Tables 1** **and** **2**). Especially, in HBMs, seven collagen components were significantly up-regulated, while matrix metalloproteinase 1 (interstitial collagenase) was dramatically up-regulated by more than 50-fold. Therefore, a reasonable explanation is that the host cells should maintain the balance of ECM synthesis and degradation. In addition, the ECM degradation may facilitate the spread of pathogenic *Leptospira* in the intercellular space. Further dynamic studies on leptospiral adhesion will uncover the function of ECM regulation during leptospiral infection.

**Table 1:** The gene regulations of ECM components, synthesis enzymes, and degrading enzymes in murine peritoneal macrophages (MPMs) after 4-h *L. interrogans* infection.

Gene and symbol	Entrez gene description	Regulation fold at 4 h
*hs3st1* (NM_010474)	heparan sulfate (glucosamine) 3-O-sulfotransferase 1	4.51
*col12a1* (NM_007730)	procollagen, type XII, alpha 1	6.13
*col5a1* (NM_015734)	procollagen, type V, alpha 1	4.39
*col18a1*, *col15a1* (NM_009929)	procollagen, type XVIII, alpha 1	3.59
*eln* (NM_007925)	Elastin	4.08
*emilin3* (NM_182840)	elastin microfibrilinterfacer 3	3.52
*lrfn5* (NM_178714)	leucine rich repeat and fibronectin type III domain containing 5	6.12
*flrt3* (NM_178382)	fibronectinleucine rich transmembrane protein 3	4.67
*fn1* (NM_010233)	fibronectin 1	−3.61
*lamb3* (NM_008484)	laminin, beta 3	3.08
*lama3* (XM_140451)	laminin, alpha 3	−4.45
*spink3* (NM_009258)	serine protease inhibitor, Kazal type 3	5.02
*spinlw1* (NM_029325)	serine protease inhibitor-like, with Kunitz and WAP domains 1 (eppin)	3.33
*mmp8* (NM_008611)	matrix metalloproteinase 8	15.94
*mmp14* (NM_008608)	matrix metalloproteinase 14 (membrane-inserted)	12.07
*mmp13* (NM_008607)	matrix metalloproteinase 13	10.85
*mmp9* (NM_013599)	matrix metalloproteinase 9	10.79
*mmp12* (NM_008605)	matrix metalloproteinase 12	6.25
*mmp3* (NM_010809)	matrix metalloproteinase 3	6.09

**Table 2:** The gene regulations of ECM components, synthesis enzymes, and degrading enzymes in human peripheral blood monocytes (HBMs) after 4-h *L. interrogans* infection.

Gene and symbol	Entrez gene description	Regulation fold at 4 h
*hs3st3a1* (NM_006042)	heparan sulfate (glucosamine) 3-O-sulfotransferase 3A1	8.06
*hs6st2* (NM_147175)	heparan sulfate 6-O-sulfotransferase 2	3.41
*hs6st3* (NM_153456)	heparan sulfate 6-O-sulfotransferase 3	3.62
*ncan* (NM_004386)	chondroitin sulfate proteoglycan 3 (neurocan)	3.38
*col22a1* (NM_152888)	collagen, type XXII, alpha 1	5.98
*col25a1* (BC036669)	collagen, type XXV, alpha 1	4.41
*col8a1* (NM_020351)	collagen, type VIII, alpha 1	3.62
*col6a1* (NM_001848)	collagen, type VI, alpha 1	3.32
*col16a1* (NM_001856)	collagen, type XVI, alpha 1	3.31
*col6a2* (NM_058174)	collagen, type VI, alpha 2	3.03
*col4a5* (NM_000495)	collagen, type IV, alpha 5 (Alport syndrome)	−3.51
*fn1* (NM_212474)	fibronectin 1	14.57
*flrt3* (NM_013281)	fibronectinleucine rich transmembrane protein 3	5.11
*spink5* (NM_006846)	serine protease inhibitor, Kazal type 5	3.71
*spink6* (NM_205841)	serine protease inhibitor, Kazal type 6	3.42
*spink1* (NM_003122)	serine protease inhibitor, Kazal type 1	3.11
*mmp1* (NM_002421)	matrix metalloproteinase 1 (interstitial collagenase)	58.89
*mmp3* (NM_002422)	matrix metalloproteinase 3 (stromelysin 1, progelatinase)	40.01
*mmp7* (NM_002423)	matrix metalloproteinase 7 (matrilysin, uterine)	8.34
*mmp9* (NM_004994)	matrix metalloproteinase 9 (gelatinase B, 92 kDa gelatinase, 92 kDa type IV collagenase)	6.75
*mmp10* (NM_002425)	matrix metalloproteinase 10 (stromelysin 2)	6.12
*mmp17* (NM_016155)	matrix metalloproteinase 17 (membrane-inserted)	4.81
*mmp19* (NM_002429)	matrix metalloproteinase 19	4.23
*mmp26* (NM_021801)	matrix metalloproteinase 26	3.81

## Discussion

Mature macrophages can phagocytize and kill pathogens, process and present antigens for the adaptive immune system, and secrete a series of cytokines and chemokines to regulate host immune response; these characteristics make macrophages an important and effective cell of host innate immunity. Macrophages express many surface receptors, such as TLRs, complement receptors, scavenger receptors, mannose receptors, etc., which help the host recognize pathogens and present antigens for adaptive immunity [76]. In this study, several differences between murine and human macrophages infected by *L. interrogans* were revealed by transcriptomics and cytokine array methods; these findings somewhat reflected the differences of chronic infection in mice and acute infection in humans.

Our previous research on *Leptospira*-macrophage interaction has shown that there is little difference of the transcriptomics of *L. interrogans* infecting murine and human macrophage cell lines [30]. In contrast, significant transcriptomics and cytokine differences of murine and human primary macrophages infected by *L. interrogans* were uncovered in this study, suggesting that different immune responses explain the major disparities in the murine and human *Leptospira*-macrophage infection models. However, live leptospiral cells can regulate their high-antigenicity antigens (such as heat-shock proteins and flagellar proteins) when they infect macrophages [35]. Hence, the different regulations of MPMs and HBMs may be partially due to the different regulations of the leptospiral genes.

The major differences of the murine and human macrophages in this study were the dramatically different expression profiles of cytokines and chemokines. These differences partially reflected the different outcomes of chronic and acute leptospiral infections [17], [19]. Considering that these cytokines can further regulate humoral immune and inflammatory responses, it is necessary to further investigate the relationship between cytokine secretion and immune response during leptospirosis on gene knockout cell or mouse models. In conclusion, this study uncovered a series of molecular changes in host immune cells, and the findings provide a foundation for further studies on different immune responses due to chronic and acute leptospiral infections.

## Supporting Information

Figure S1 **Efficiencies of leptospiral infection revealed using indirect immunofluorescences of leptospiral cells and macrophage lysosomes.** Colocalization of *Leptospira* and lysosome after 1-h infection were used to verify the efficiencies of leptospiral infection. A and C: MPMs not infected by *Leptospira*, in which lysosomes were revealed by green fluorescence labeling of Lamp 1 marker; B and D: MPMs infected by *L. biflexa* and *L. interrogans*, respectively. Leptospiral cells were labeled with red fluorescence, and the yellow fluorescence indicated the phagolysosomes. E and G: HBMs not infected by *Leptospira*, in which lysosomes were revealed by green fluorescence labeling of Lamp 1 marker. F and H: HBMs infected by *L. biflexa* and *L. interrogans*, respectively. Leptospiral cells were labeled with red fluorescence, and the yellow fluorescence indicated the phagolysosome. The scale bars in the figures correspond to 20 µm.
(TIF)

Figure S2 **RNA integrities of macrophages verified using high performance capillary electrophoresis (HPCE).** The panels of MPM-0/1/2/4 show the RNA samples of uninfected MPMs and MPMs infected by *L. interrogans* for 1-, 2-, and 4-h, respectively. The panels of HBM-0/1/2/4 show the RNA samples of uninfected HBMs and HBMs infected by *L. interrogans* for 1-, 2-, and 4-h, respectively. The last panel shows the RNA 6000 Nano ladder, which contains six RNA fragments ranging in size from 0.2 to 6 kb (0.2 kb, 0.5 kb, 1.0 kb, 2.0 kb, 4.0 kb, and 6.0 kb) at a total concentration of 150 ng/µl.
(TIF)

Figure S3 **Validation of the microarray data of murine peritoneal macrophages (MPMs) infected by** ***L. interrogans*** **using qRT-PCR.** MPM 1/0, MPM 2/0, and MPM 4/0 indicate the gene expression fold change at 1-, 2-, and 4-h, respectively. The qRT-PCR values were plotted against the microarray data values. The high correlation coefficient values (R^2^≥0.85) indicated that the microarray signal represented by multiple oligonucleotide probes was valid for transcriptomics research.
(TIF)

Figure S4 **Validation of the microarray data of human peripheral blood monocytes (HBMs) infected by** ***L. interrogans*** **using qRT-PCR.** HBM 1/0, HBM 2/0, and HBM 4/0 indicate the gene expression fold change at 1-, 2-, and 4-h, respectively. The qRT-PCR values were plotted against the microarray data values. The high correlation coefficient values (R^2^≥0.85) indicated that the microarray signal represented by multiple oligonucleotide probes was valid for transcriptomics research.
(TIF)

Figure S5 **Verification of highly regulated genes and differentially expressed genes using qRT-PCR.** The gene regulations of sixteen highly regulated genes after 4-h infections, such as TNF-alpha, IL-6, CASP8 and FADD-like apoptosis regulator, IL-1-alpha, IL-1-beta, TLR1, LPS-binding protein, NF-kappa B inhibitor alpha, IL-10, CCL5, CXCL9, MIP-1 alpha, MIP-1 beta, MIP-2, C3, and BCL-2, were further verified by qRT-PCR. Three biological replicates were designed for each cell infection model, and a new batch of RNA samples were used for qRT-PCR. The pathogenic *Leptospira*-specific gene regulations were labeled with column shadows.
(PPT)

Table S1 **Primers for verification of murine peritoneal macrophage (MPM) microarray data using qRT-PCR.**
(DOC)

Table S2 **Primers for verification of human peripheral blood monocyte (HBM) microarray data using qRT-PCR.**
(DOC)

Table S3 **Primers for verification of highly regulated genes and differentially expressed genes using qRT-PCR.**
(XLS)
