# Supplementary figures and images for: Responses of Murine and Human Macrophages to Leptospiral Infection: A Study Using Comparative Array Analysis
Source: PLoS Negl Trop Dis. 2013 Oct 10;7(10):e2477. doi: 10.1371/journal.pntd.0002477 (PMC3794915; doi:10.1371/journal.pntd.0002477)

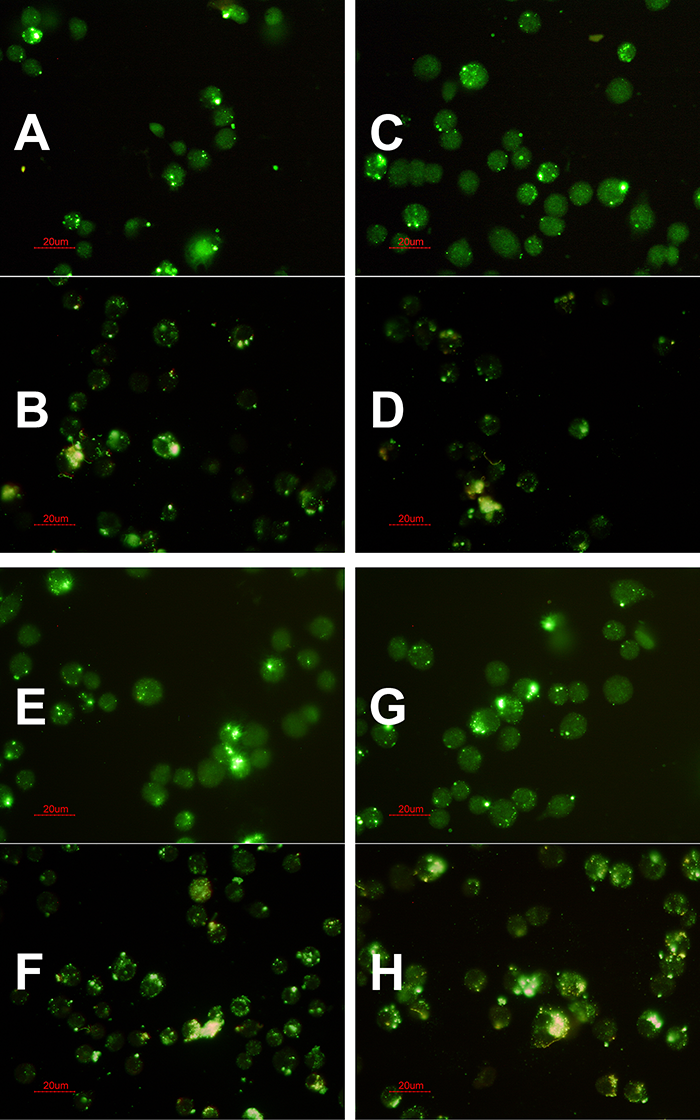

Supplement: Figure S1 — Efficiencies of leptospiral infection revealed using indirect immunofluorescences of leptospiral cells and macrophage lysosomes. Colocalization of Leptospira and lysosome after 1-h infection were used to verify the efficiencies of leptospiral infection. A and C: MPMs not infected by Leptospira, in which lysosomes were revealed by green fluorescence labeling of Lamp 1 marker; B and D: MPMs infected by L. biflexa and L. interrogans, respectively. Leptospiral cells were labeled with red fluorescence, and the yellow fluorescence indicated the phagolysosomes. E and G: HBMs not infected by Leptospira, in which lysosomes were revealed by green fluorescence labeling of Lamp 1 marker. F and H: HBMs infected by L. biflexa and L. interrogans, respectively. Leptospiral cells were labeled with red fluorescence, and the yellow fluorescence indicated the phagolysosome. The scale bars in the figures correspond to 20 µm. (TIF) [file pntd.0002477.s001.tif]

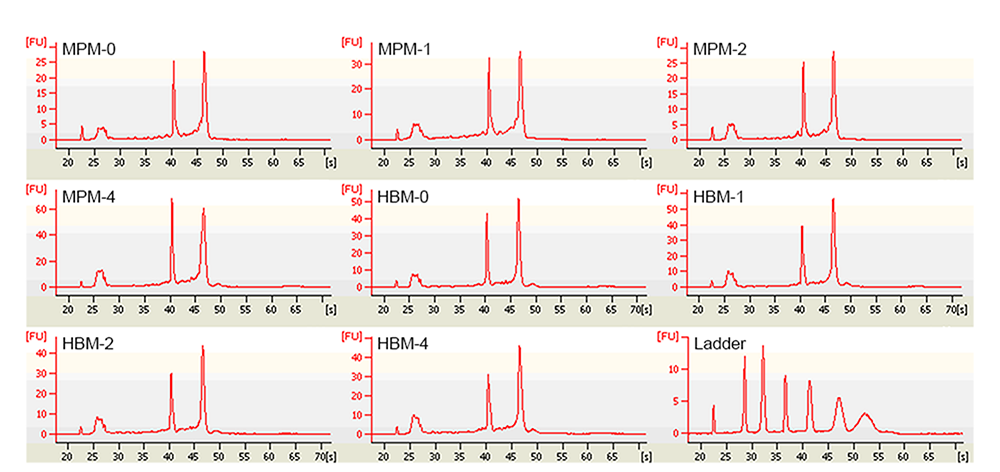

Supplement: Figure S2 — RNA integrities of macrophages verified using high performance capillary electrophoresis (HPCE). The panels of MPM-0/1/2/4 show the RNA samples of uninfected MPMs and MPMs infected by L. interrogans for 1-, 2-, and 4-h, respectively. The panels of HBM-0/1/2/4 show the RNA samples of uninfected HBMs and HBMs infected by L. interrogans for 1-, 2-, and 4-h, respectively. The last panel shows the RNA 6000 Nano ladder, which contains six RNA fragments ranging in size from 0.2 to 6 kb (0.2 kb, 0.5 kb, 1.0 kb, 2.0 kb, 4.0 kb, and 6.0 kb) at a total concentration of 150 ng/µl. (TIF) [file pntd.0002477.s002.tif]

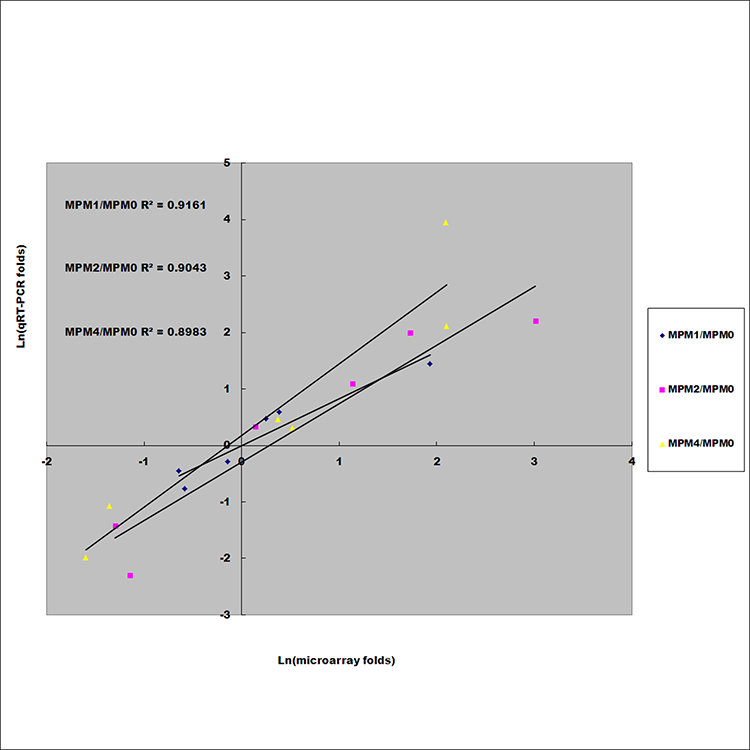

Supplement: Figure S3 — Validation of the microarray data of murine peritoneal macrophages (MPMs) infected by L. interrogans using qRT-PCR. MPM 1/0, MPM 2/0, and MPM 4/0 indicate the gene expression fold change at 1-, 2-, and 4-h, respectively. The qRT-PCR values were plotted against the microarray data values. The high correlation coefficient values (R2≥0.85) indicated that the microarray signal represented by multiple oligonucleotide probes was valid for transcriptomics research. (TIF) [file pntd.0002477.s003.tif]

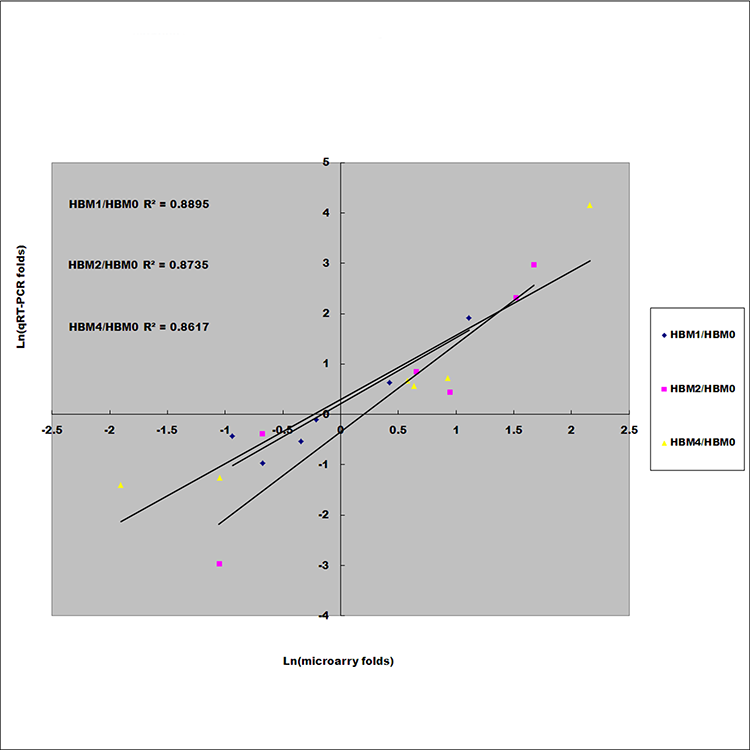

Supplement: Figure S4 — Validation of the microarray data of human peripheral blood monocytes (HBMs) infected by L. interrogans using qRT-PCR. HBM 1/0, HBM 2/0, and HBM 4/0 indicate the gene expression fold change at 1-, 2-, and 4-h, respectively. The qRT-PCR values were plotted against the microarray data values. The high correlation coefficient values (R2≥0.85) indicated that the microarray signal represented by multiple oligonucleotide probes was valid for transcriptomics research. (TIF) [file pntd.0002477.s004.tif]
