# Supplementary material for: Responses of Murine and Human Macrophages to Leptospiral Infection: A Study Using Comparative Array Analysis
Source: PLoS Negl Trop Dis. 2013 Oct 10;7(10):e2477. doi: 10.1371/journal.pntd.0002477 (PMC3794915; doi:10.1371/journal.pntd.0002477)
Supplement: Figure S5 — Verification of highly regulated genes and differentially expressed genes using qRT-PCR. The gene regulations of sixteen highly regulated genes after 4-h infections, such as TNF-alpha, IL-6, CASP8 and FADD-like apoptosis regulator, IL-1-alpha, IL-1-beta, TLR1, LPS-binding protein, NF-kappa B inhibitor alpha, IL-10, CCL5, CXCL9, MIP-1 alpha, MIP-1 beta, MIP-2, C3, and BCL-2, were further verified by qRT-PCR. Three biological replicates were designed for each cell infection model, and a new batch of RNA samples were used for qRT-PCR. The pathogenic Leptospira-specific gene regulations were labeled with column shadows. (PPT) [file pntd.0002477.s005.ppt]

## Slide 1
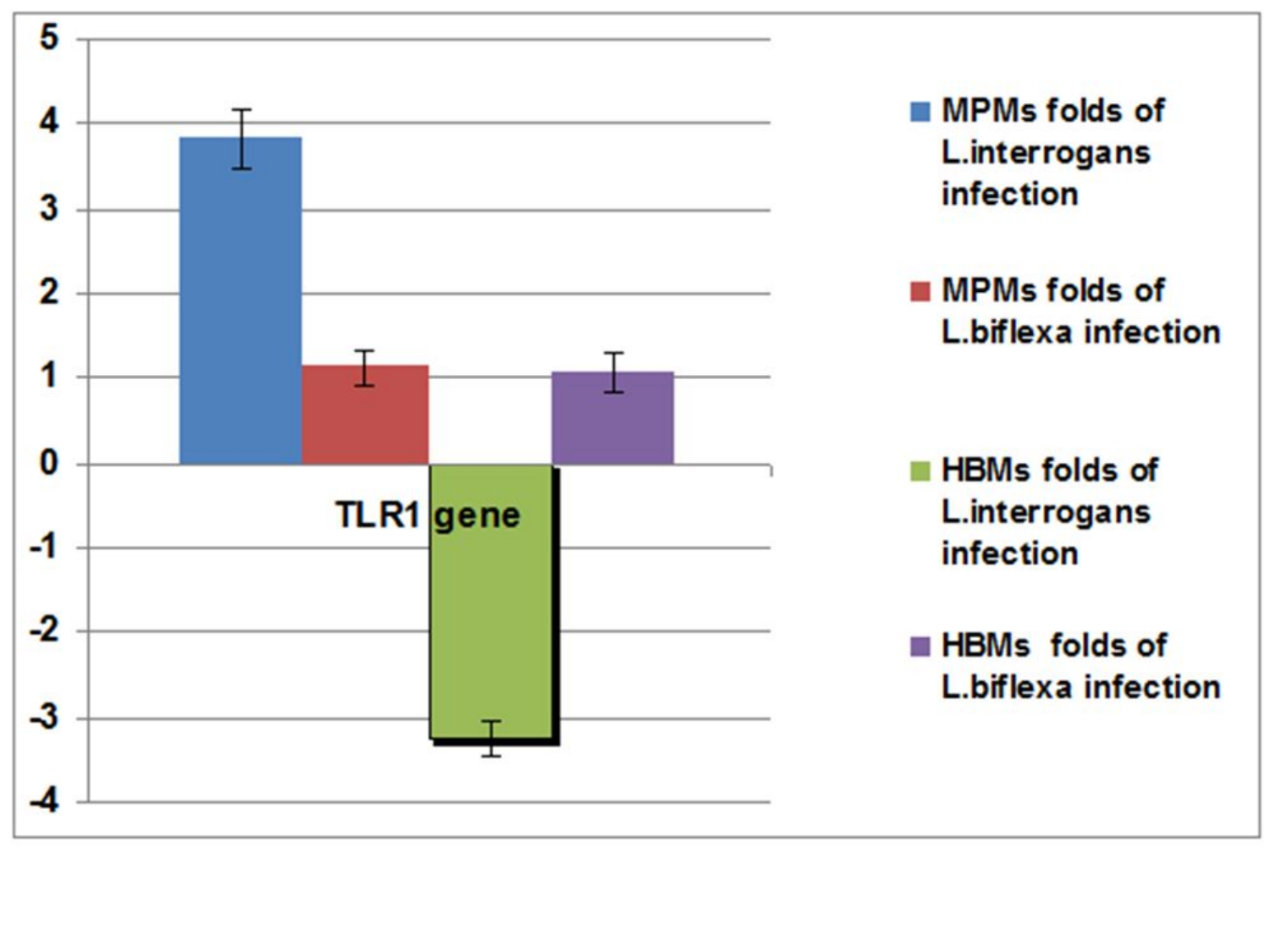

#

## Slide 2
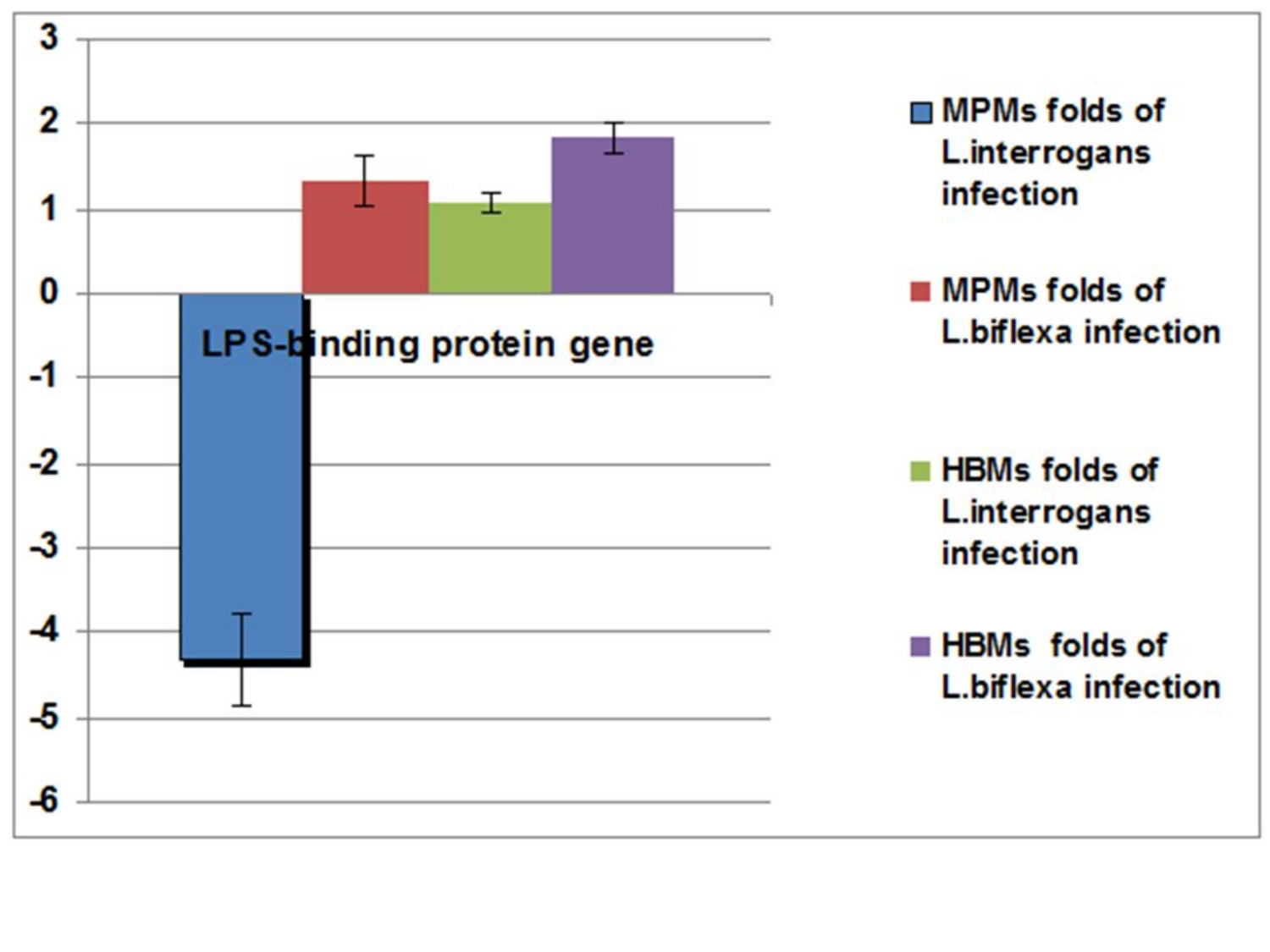

#

## Slide 3
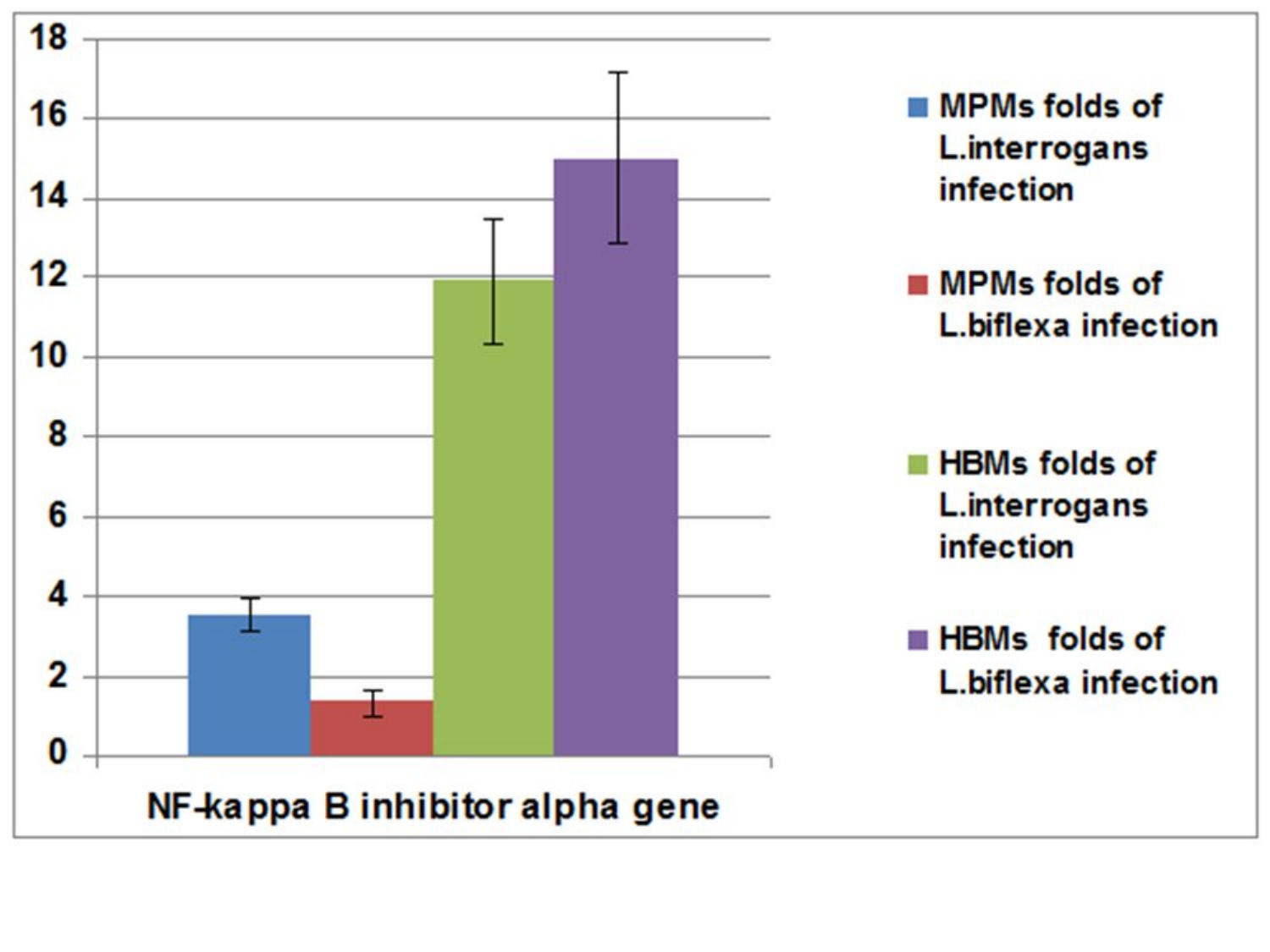

#

## Slide 4
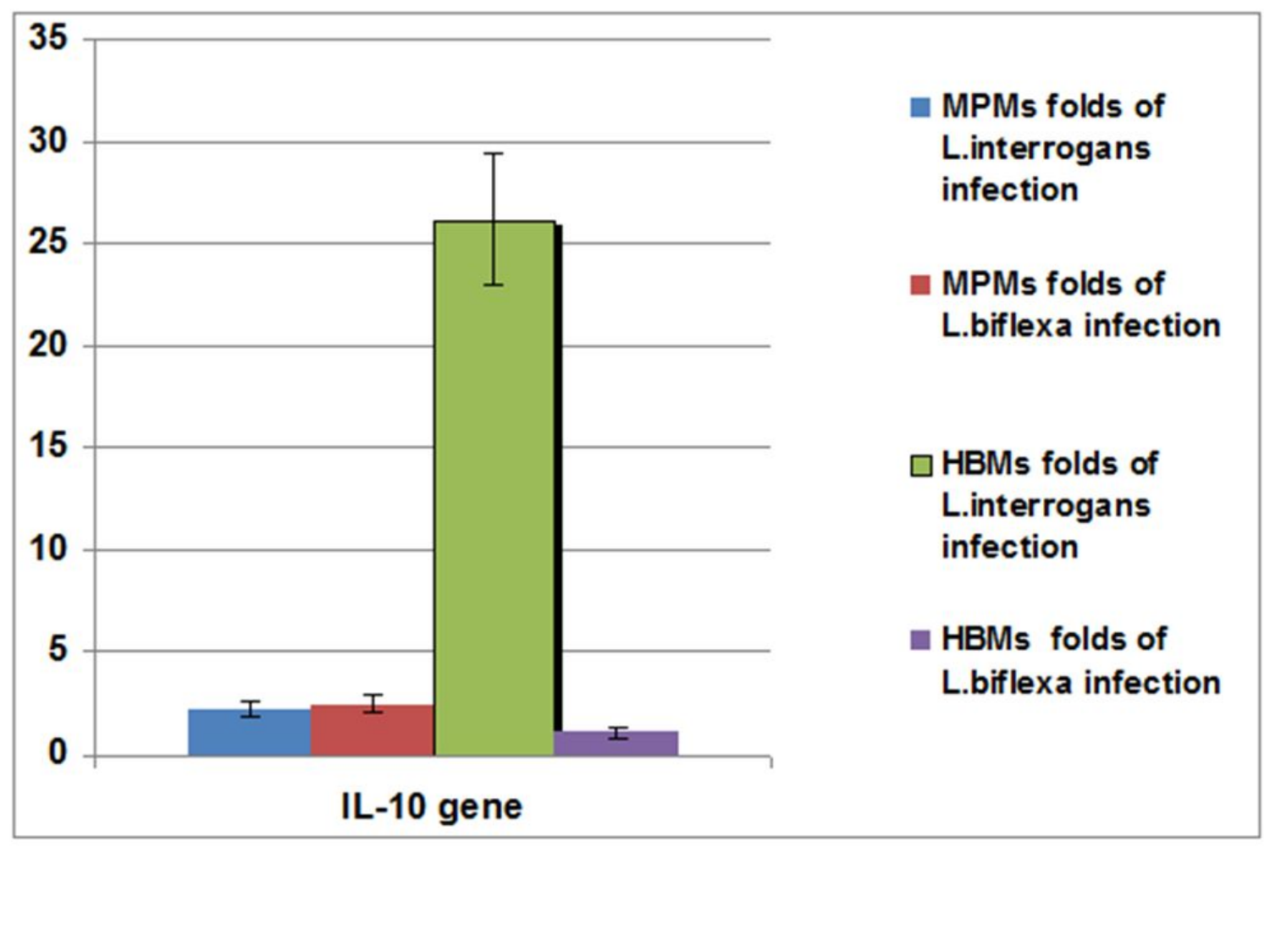

#

## Slide 5
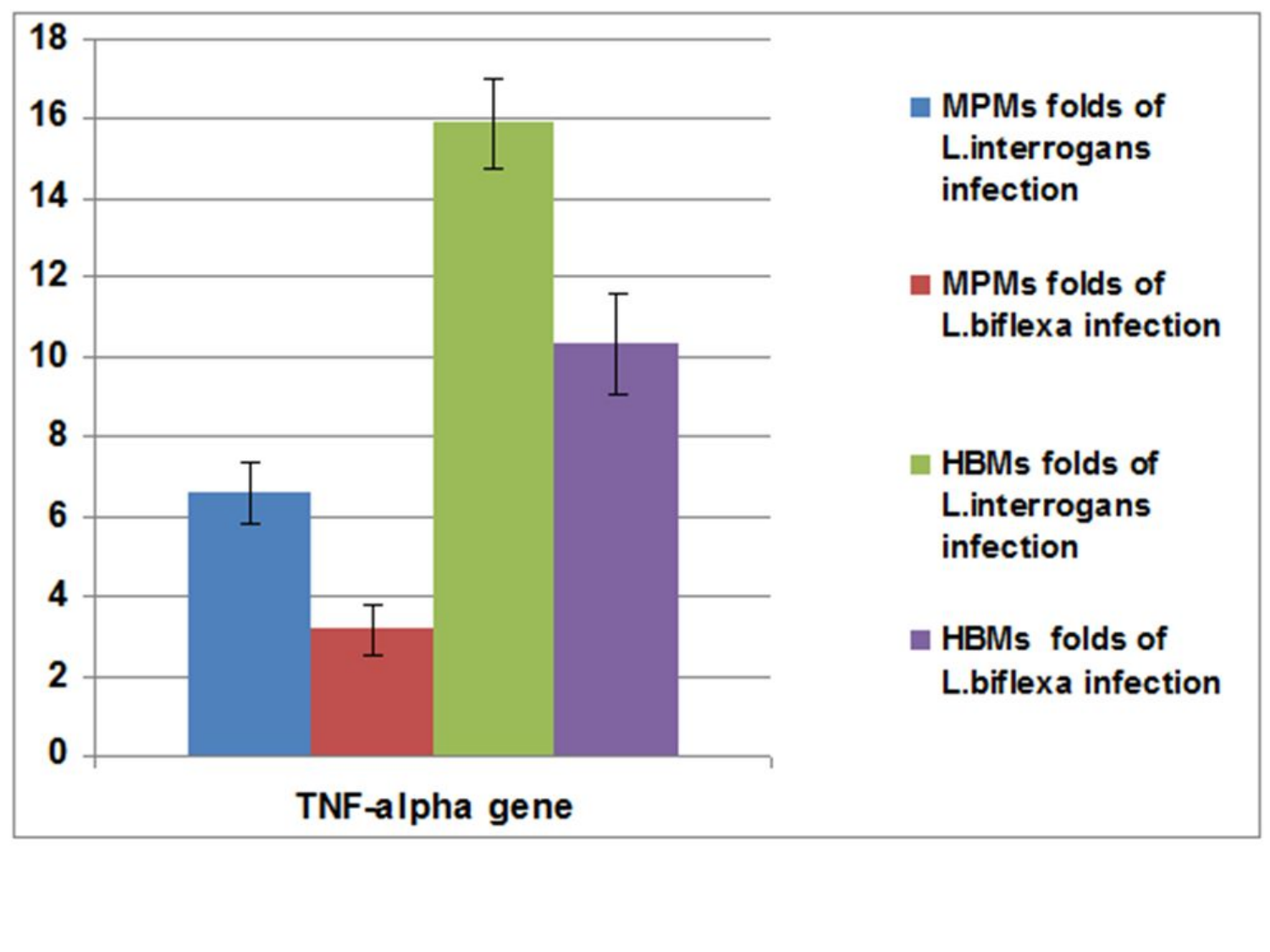

#

## Slide 6
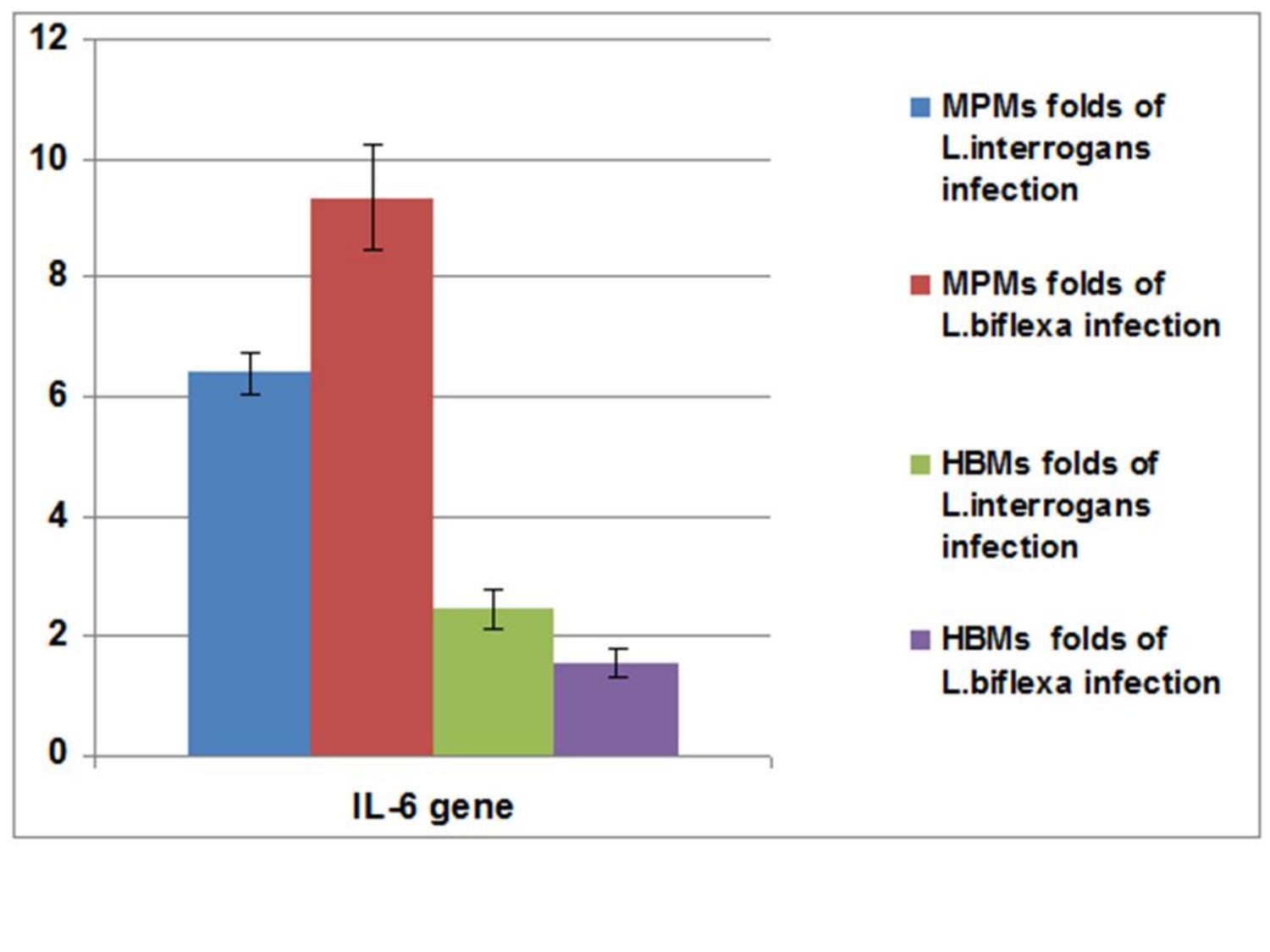

#

## Slide 7
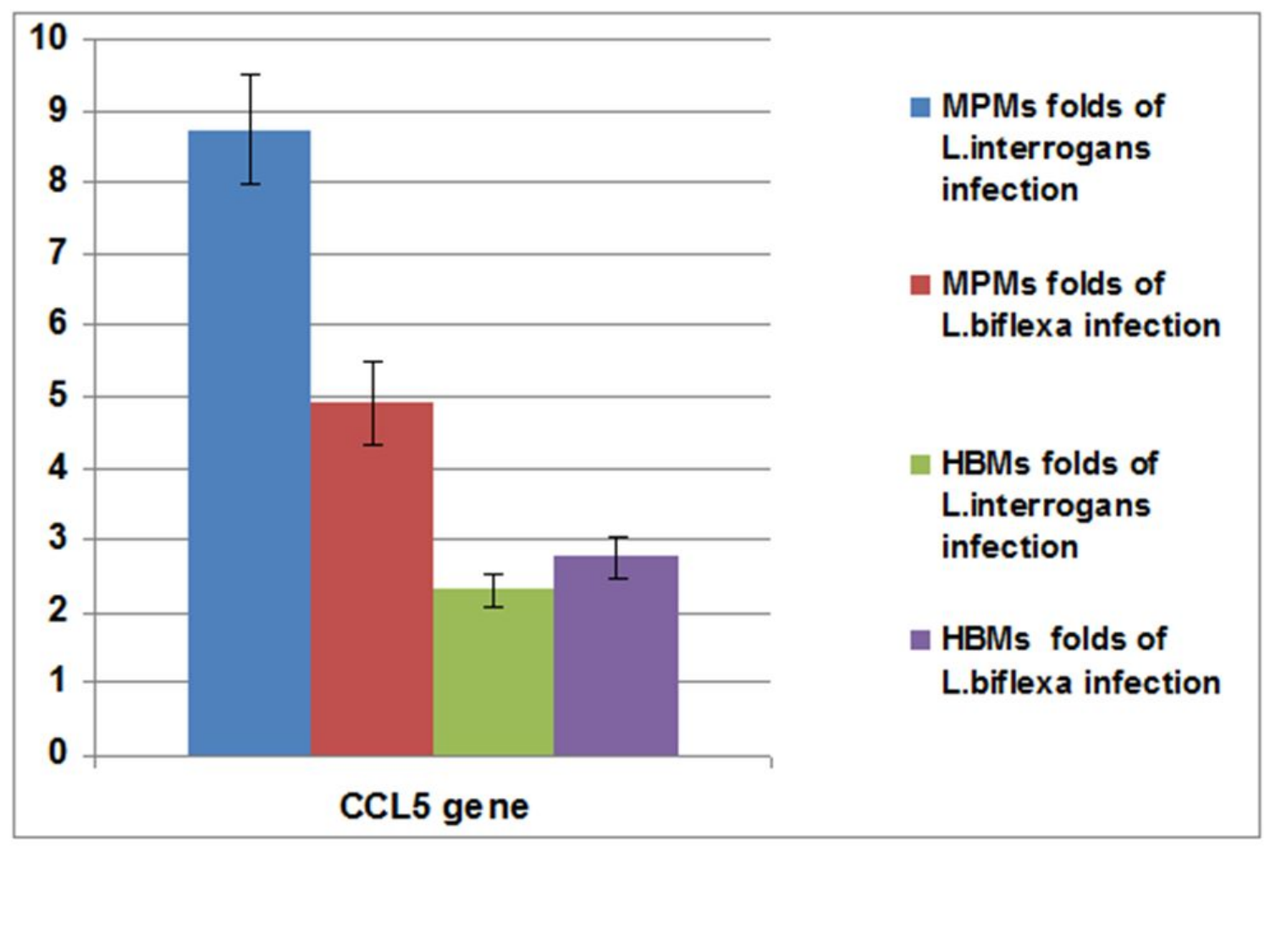

#

## Slide 8
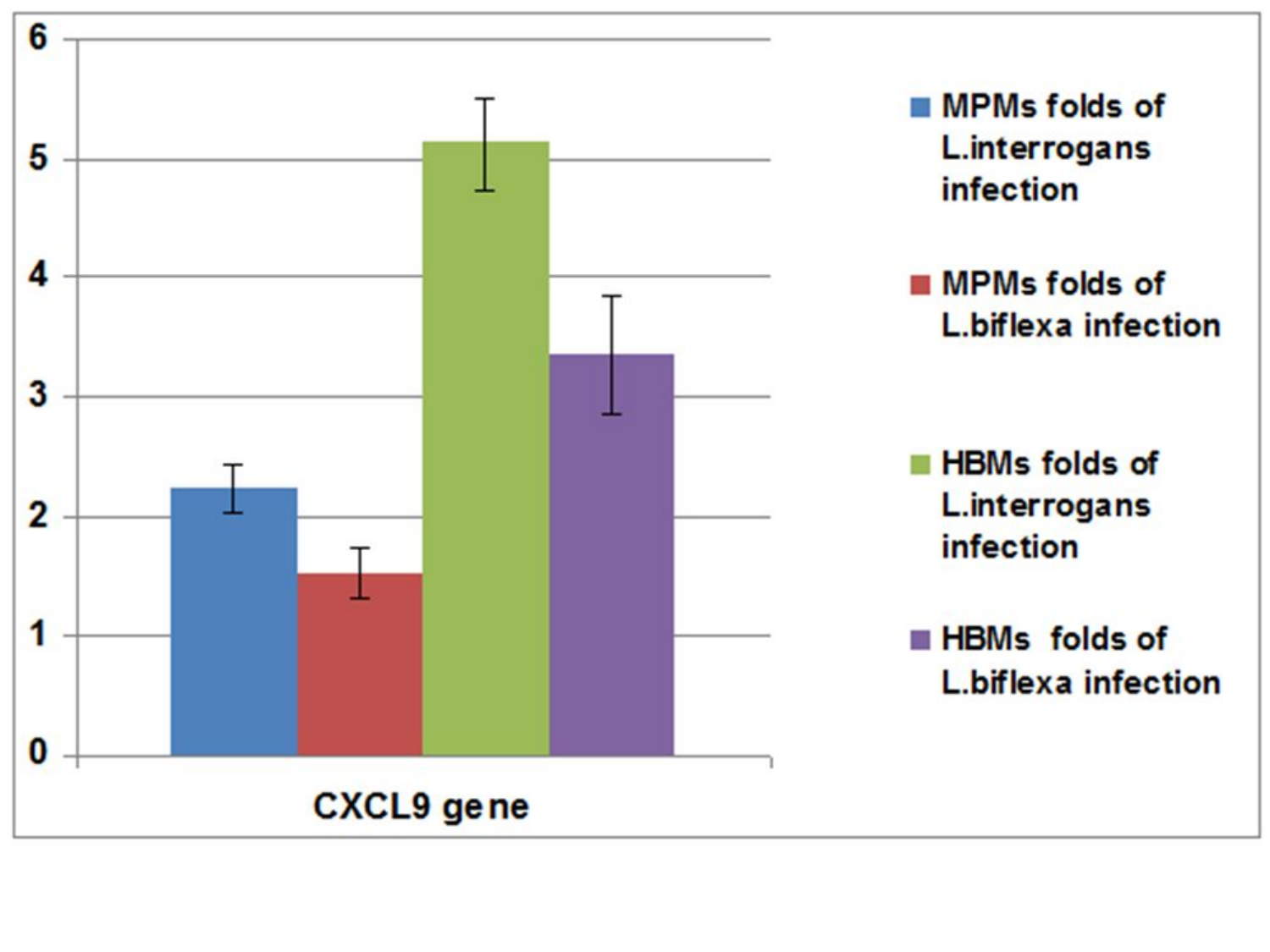

#

## Slide 9
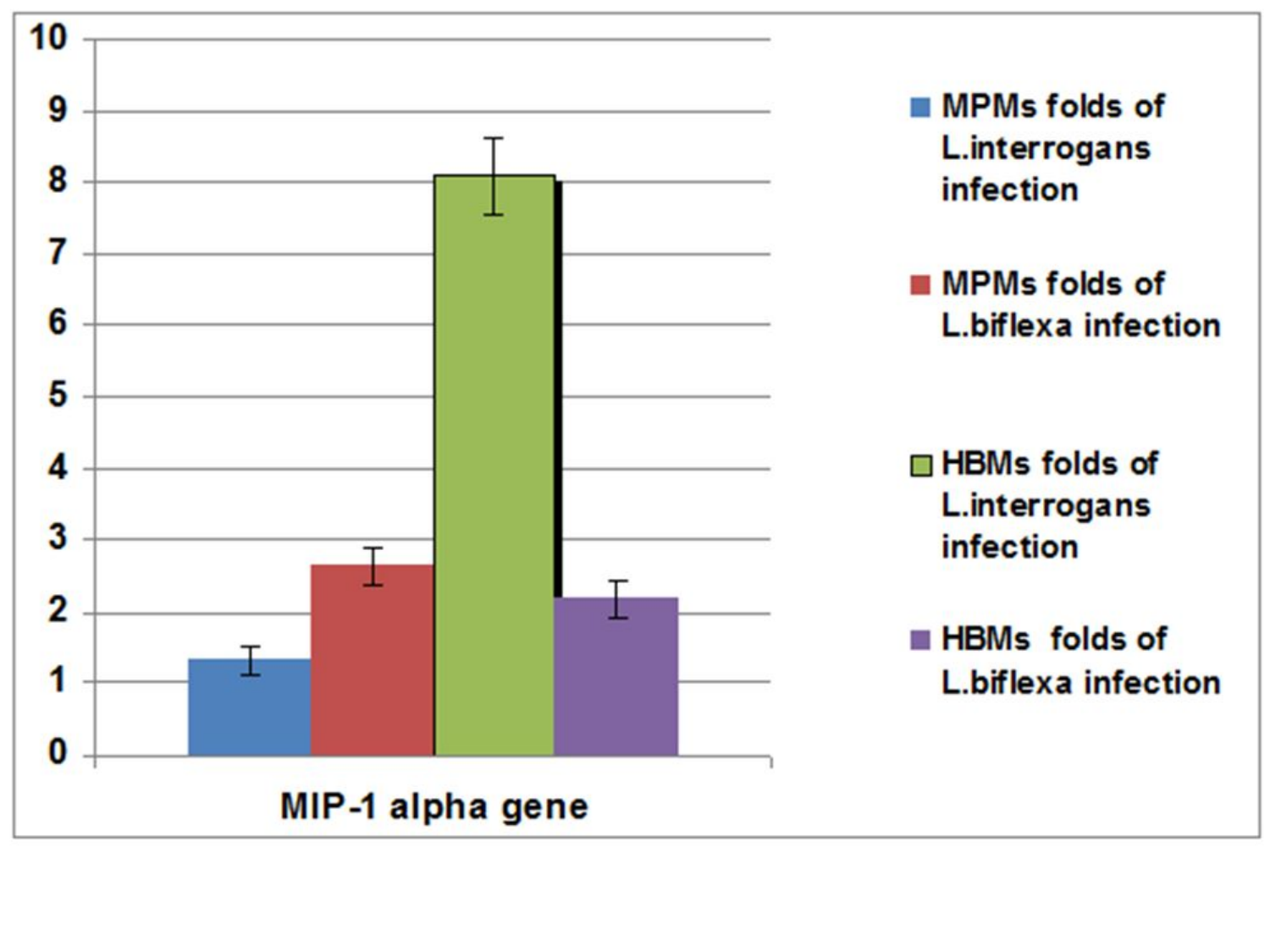

#

## Slide 10
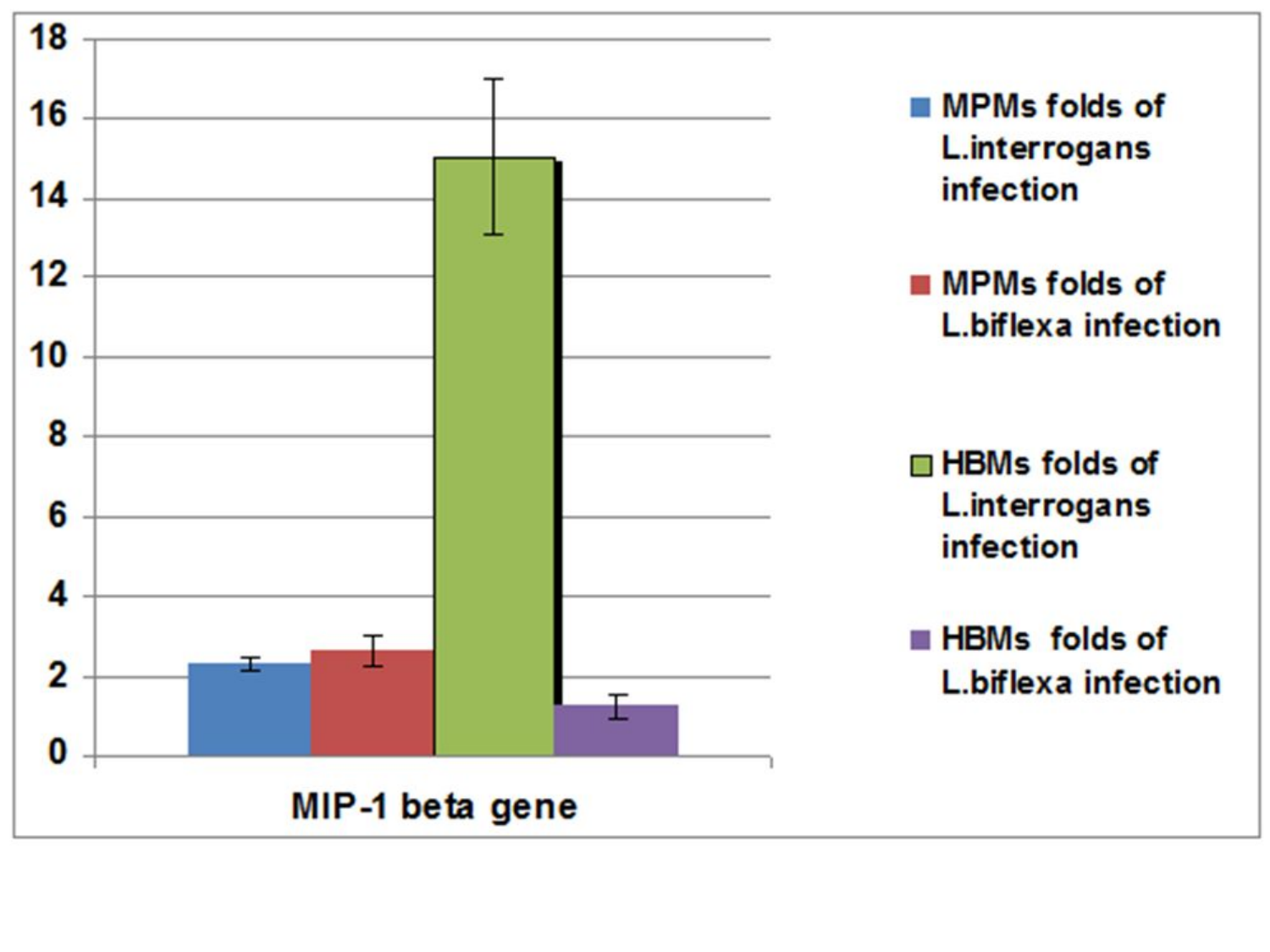

#

## Slide 11
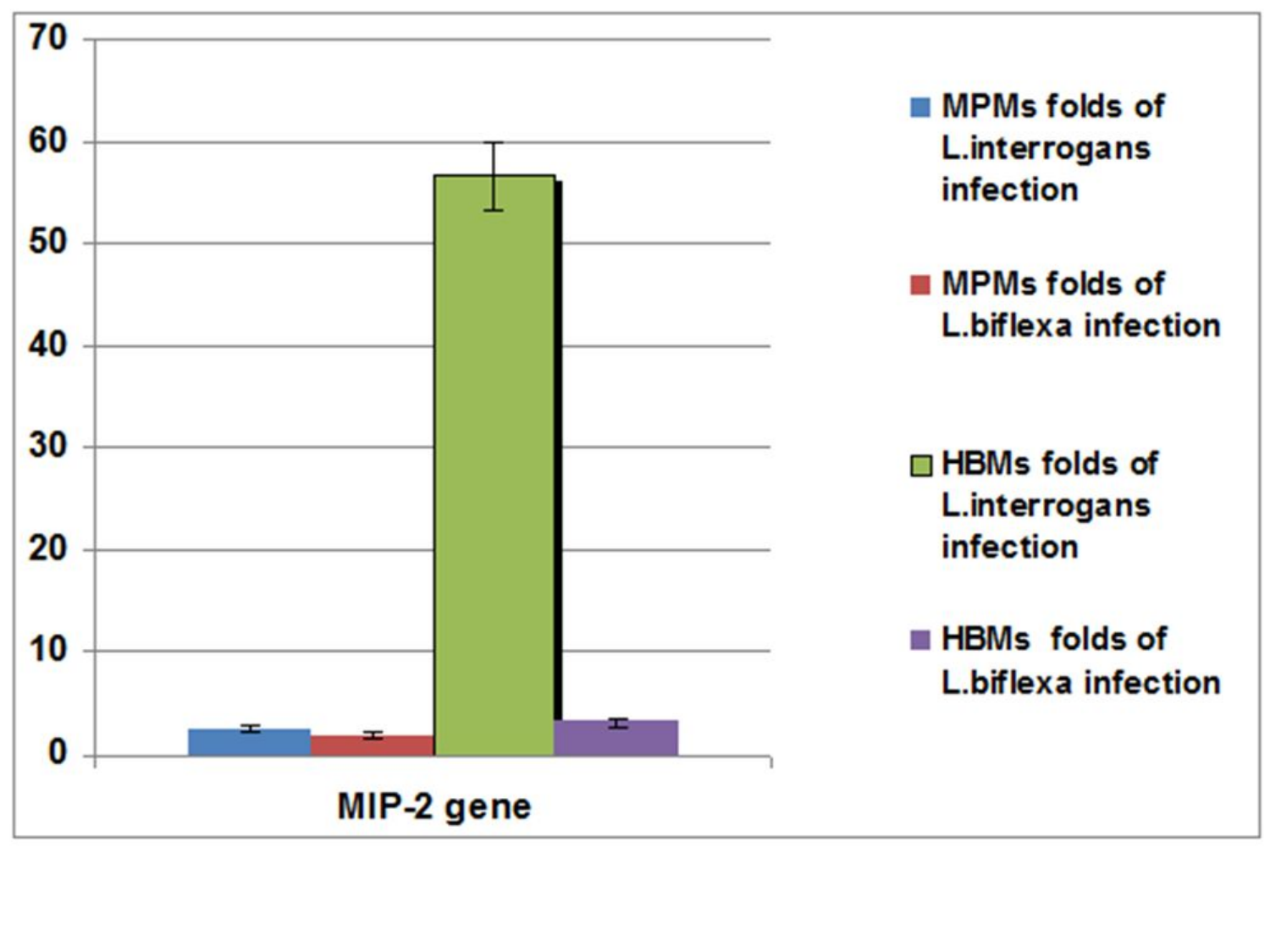

#

## Slide 12
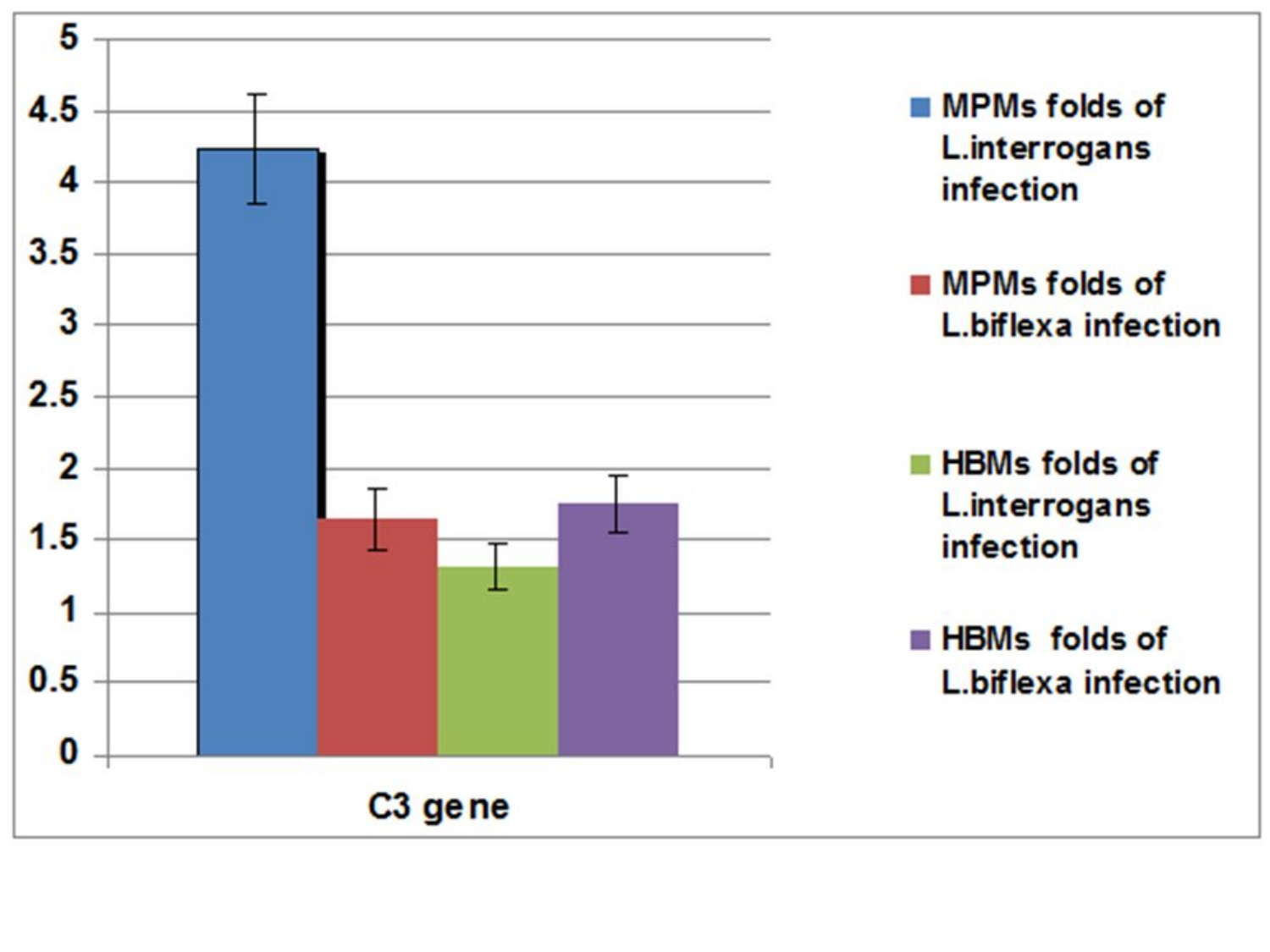

#

## Slide 13
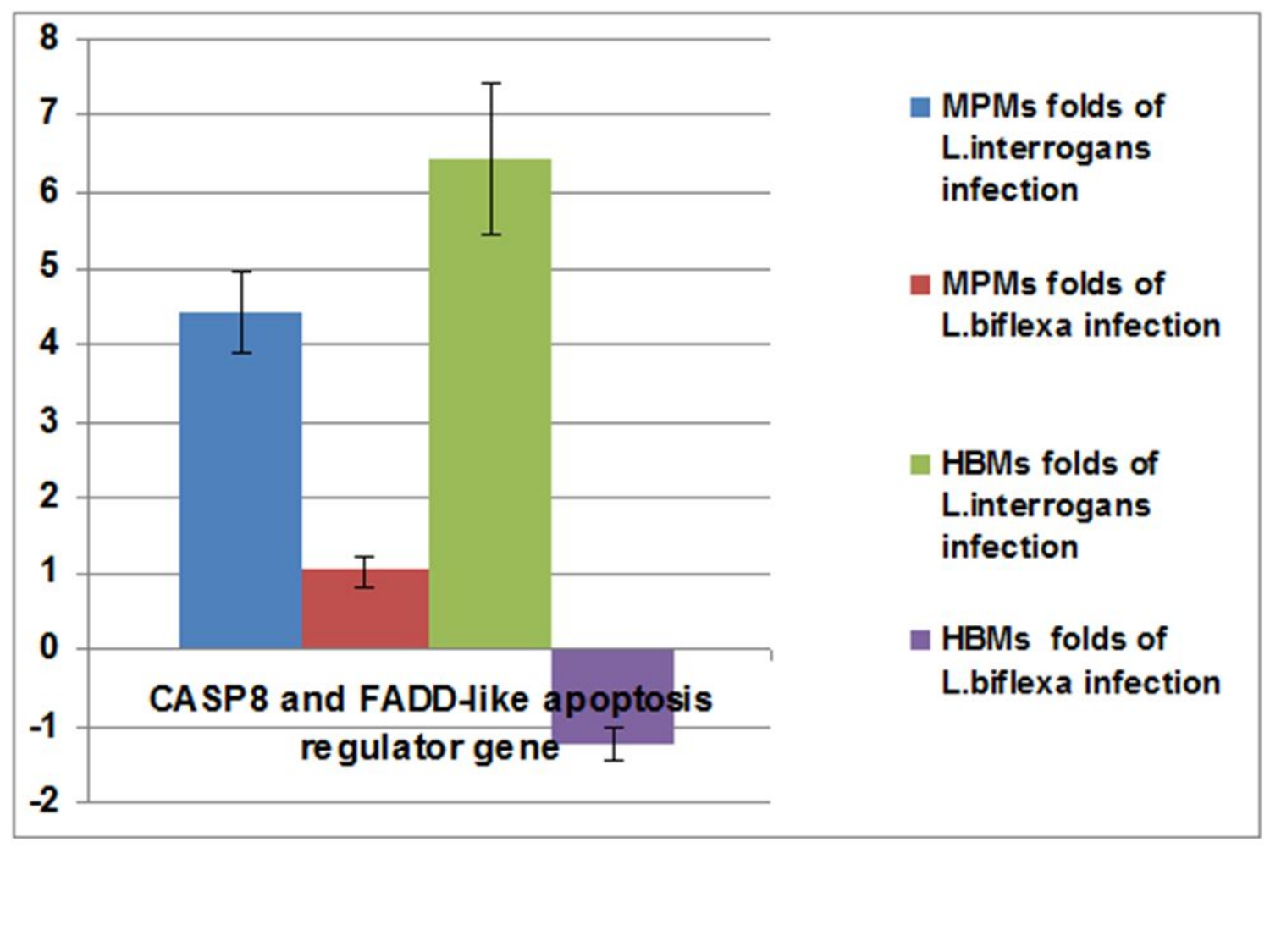

#

## Slide 14
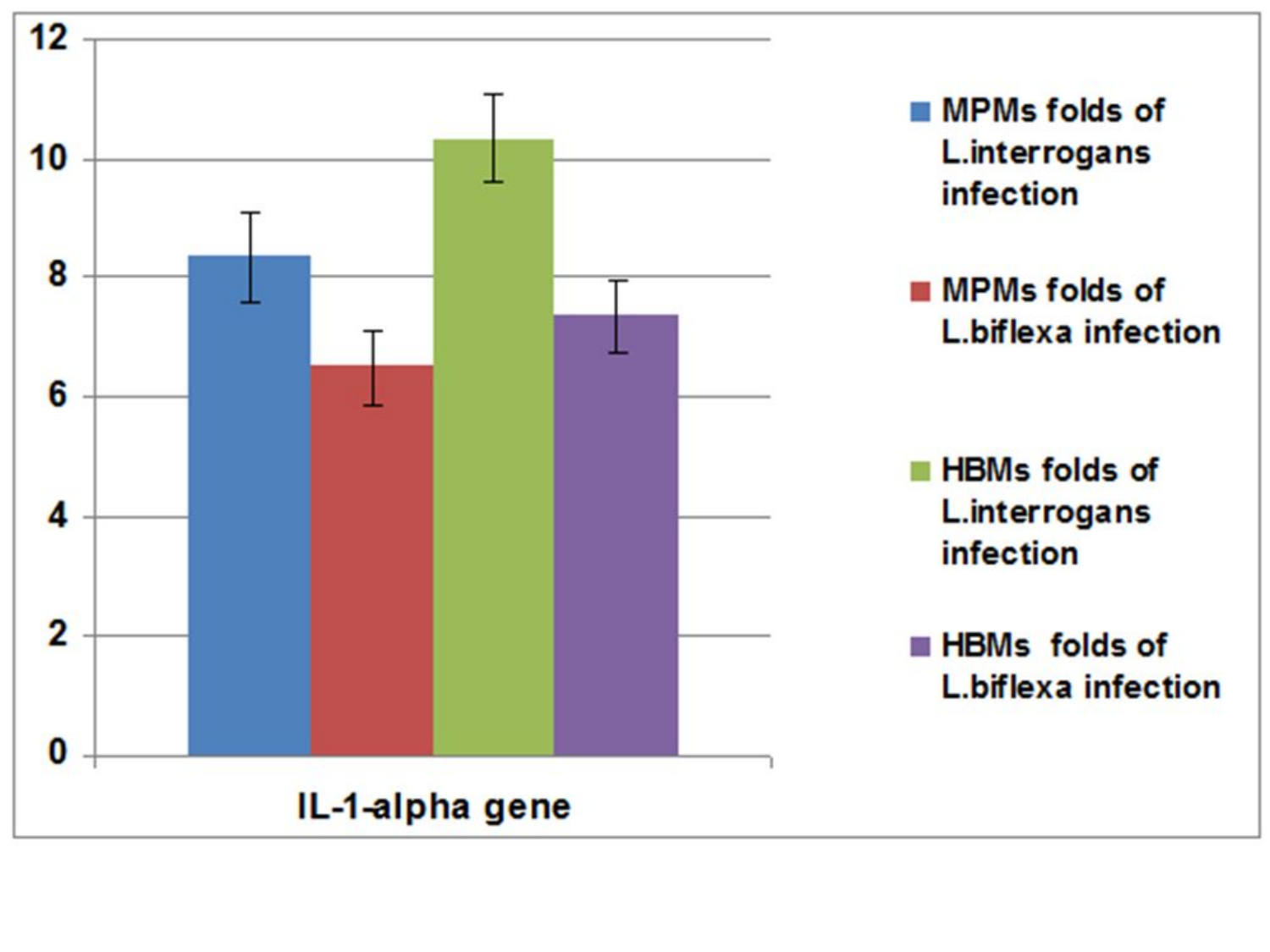

#

## Slide 15
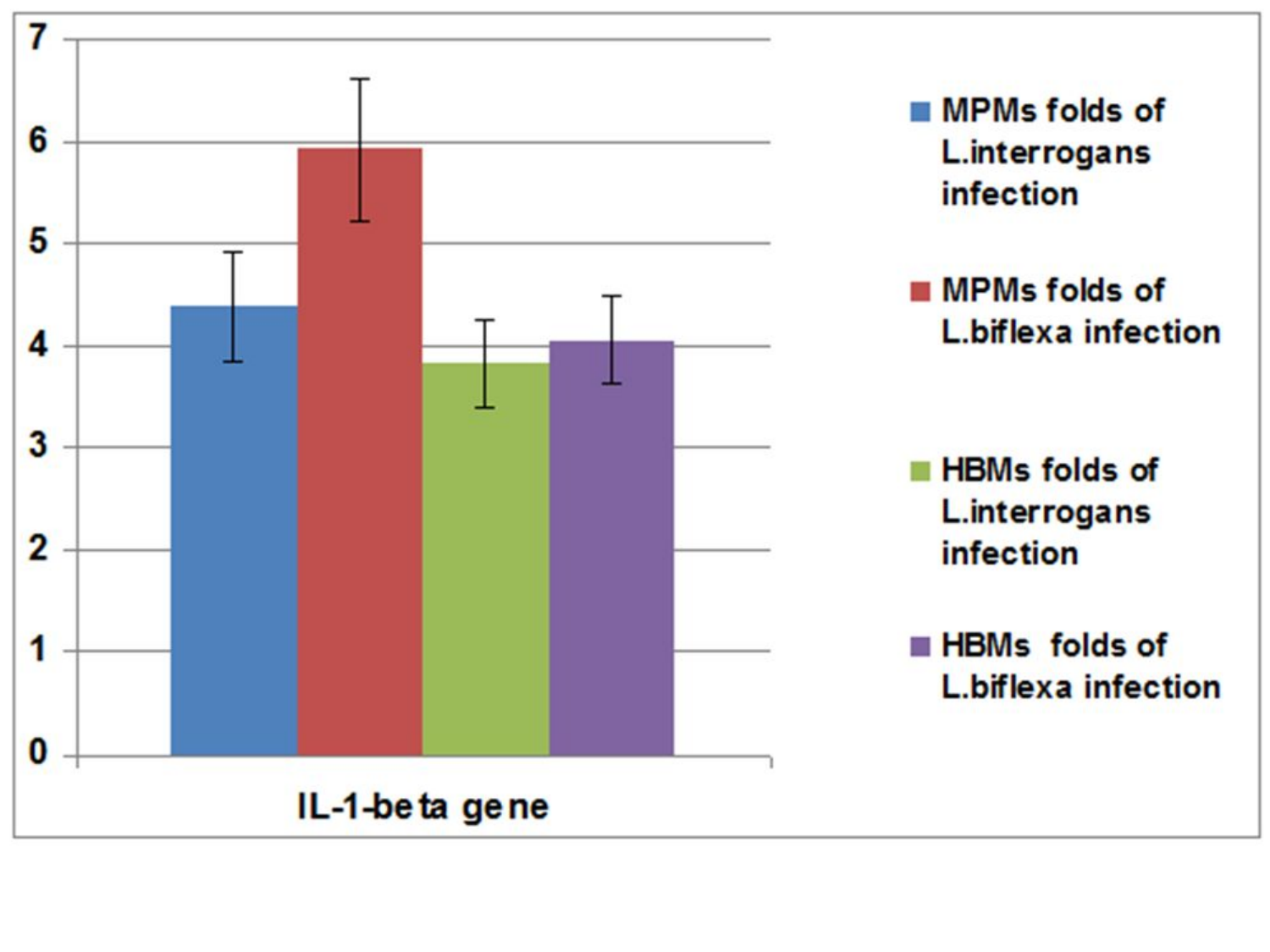

#

## Slide 16
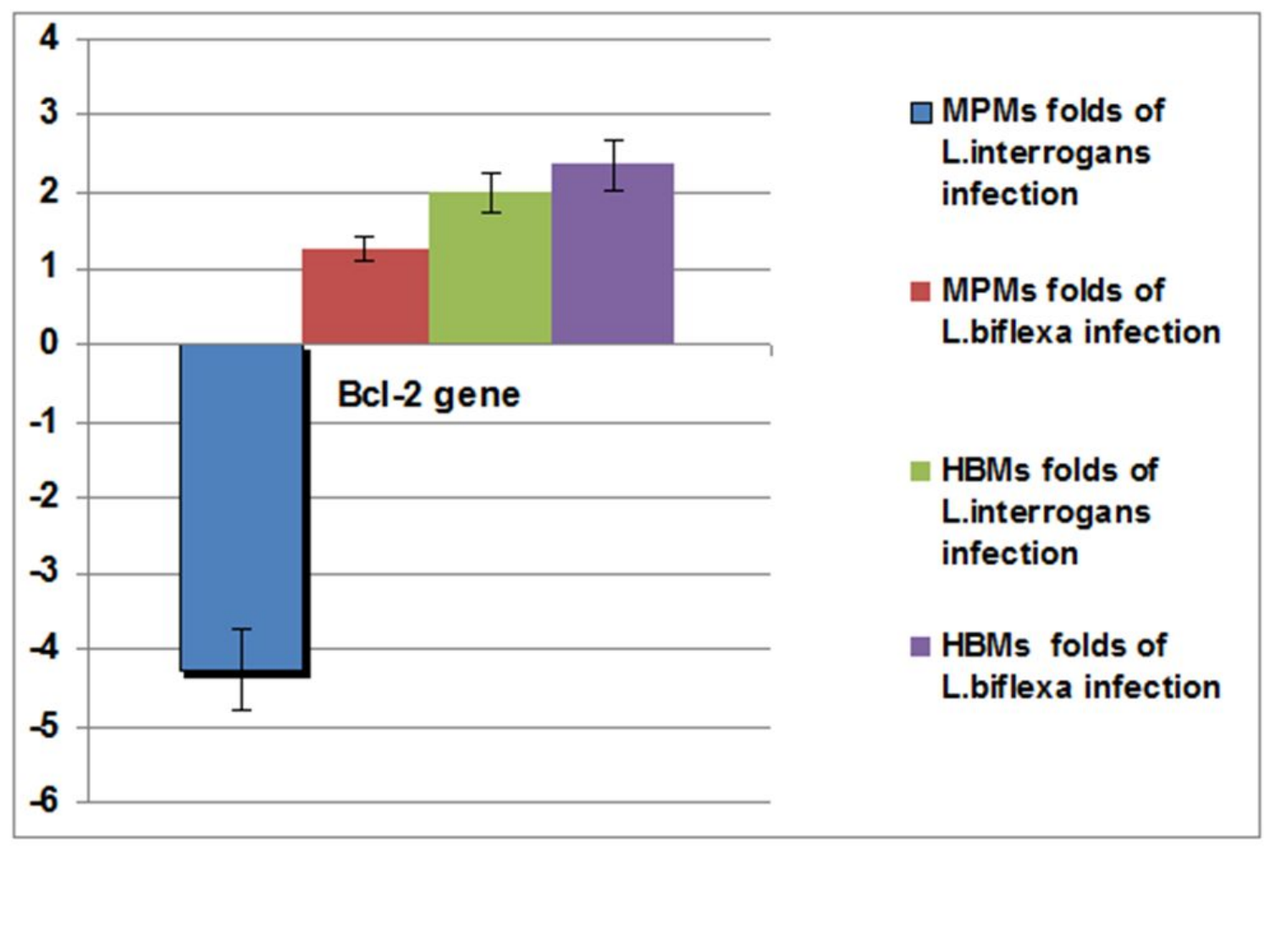

#
