# Supplementary material for: Responses of Murine and Human Macrophages to Leptospiral Infection: A Study Using Comparative Array Analysis
Source: PLoS Negl Trop Dis. 2013 Oct 10;7(10):e2477. doi: 10.1371/journal.pntd.0002477 (PMC3794915; doi:10.1371/journal.pntd.0002477)
Supplement: Table S1 — Primers for verification of murine peritoneal macrophage (MPM) microarray data using qRT-PCR. (DOC) [file pntd.0002477.s006.doc]

**Table S1. Primers for verification of murine peritoneal macrophage (MPM) microarray data using qRT-PCR.**

| Gene and Symble | Forward primer sequence | Reverse primer sequence | Amplicom size (bp) | Annealing temp. (℃) |
| --- | --- | --- | --- | --- |
| *rasa4* (NM_133914.2) | CTTGAGGCGAGGGACTTAGCA | CAGCGTGGGTAGCAGGATTTC | 122 | 56.2 |
| *f3* (NM_010171.3) | TCATCATTGTGGGAGCAGTGGTG | AGCGCGACGGGGTTCTT | 120 | 56.6 |
| *gnb5* (NM_010313.2) | ACCTGGGTGATGGCGTGTGCTTAC | GGCGGCCATGTTCTCGTTCTTG | 117 | 57.4 |
| *blm* (NM_007550.4) | CCTGTGGGGCATCCTAATAAAGAG | TCGGGGTGGCTGAGAATCCTGT | 118 | 54.2 |
| *ctsf* (NM_019861.1) | GGAGCTGAGCCGGAATGAAAATAA | GAGGGGCCGGAATGGGTGAGCA | 127 | 57.2 |
| *lbp* (NM_008489.2) | ATCACCGCTCTCCAGTTGCTACCC | ACCCGGCTGGCTATGTTGAAGG | 112 | 56.2 |
