# Supplementary material for: Responses of Murine and Human Macrophages to Leptospiral Infection: A Study Using Comparative Array Analysis
Source: PLoS Negl Trop Dis. 2013 Oct 10;7(10):e2477. doi: 10.1371/journal.pntd.0002477 (PMC3794915; doi:10.1371/journal.pntd.0002477)
Supplement: Table S2 — Primers for verification of human peripheral blood monocyte (HBM) microarray data using qRT-PCR. (DOC) [file pntd.0002477.s007.doc]

**Table S2. Primers for verification of human peripheral blood monocyte (HBM) microarray data using qRT-PCR.**

| Gene and Symble | Forward primer sequence | Reverse primer sequence | Amplicom size (bp) | Annealing temp. (℃) |
| --- | --- | --- | --- | --- |
| *st8sia2* (NM_006011.3) | GGCGGCAGAACCATTAACCCTACA | ACGCAAATCCCCAATCACAGACTT | 116 | 54.5 |
| *dusp14* (NM_007026.2) | CTCCCCGCCGTCTGCTCCCTCTCC | ATGTCCCCTCCCTCCCTATCTCTT | 120 | 54.5 |
| *itgb2* (NM_000211.3) | GCCGTGGGGGAGCTGTCTGAG | GTGTCGGGGAGGGCGTTGTGAT | 110 | 57.2 |
| *gna15* (NM_002068.2) | CCCGAGGGCAGCAAGAAG | GGGCGAGCACCGAGTCC | 118 | 57.7 |
| *p2ry5* (NM_005767.5) | CCTCGTGGCACATTCCCTCAT | AATTTTGTTGCCAGTTGTTTCTAC | 119 | 54.0 |
| *aprt* (NM_000485.2) | GACTGGGCTGCGTGCTCATCC | TGGCTCCAGGGCGTCTTTCT | 119 | 58.7 |
